# The Effect of Intravenous Dextrose Administration on Postoperative Nausea and Vomiting: Systematic Review and Meta-Analysis with Trial Sequential Analysis

**DOI:** 10.3390/medicina62091718

**Published:** 2026-09-07

**Authors:** Jae Hyuk Choi, Pyung Gul Park, Geun Joo Choi, Hyun Kang

**Affiliations:** 1 Department of Anesthesiology and Pain Medicine, Chung-Ang University Hospital, 102 Heukseok-ro, Dongjak-gu, Seoul 06973, Republic of Korea; 07693@cauhs.or.kr (J.H.C.); pistis23@cau.ac.kr (G.J.C.); 2 Department of Anesthesiology and Pain Medicine, Chung-Ang University College of Medicine, 84 Heukseok-ro, Dongjak-gu, Seoul 06911, Republic of Korea; ppgifyou@naver.com

**Keywords:** antiemetics, glucose, infusions, intravenous, meta-analysis, perioperative care, postoperative nausea and vomiting

## Abstract

*Background and Objectives*: Intravenous dextrose administration is a potential nonpharmacological adjunct for preventing postoperative nausea and vomiting (PONV); however, its efficacy and optimal timing remain unclear. This systematic review and meta-analysis, including trial sequential analysis (TSA), evaluated the antiemetic effects of perioperative dextrose administration. *Materials and Methods*: Electronic databases were searched for randomized controlled trials (RCTs) comparing dextrose-containing fluids with dextrose-free solutions under general anesthesia. The primary outcomes were the incidence of postoperative nausea (PON) and vomiting (POV) and PONV during the early (0–6 h) and late (6–48 h) periods, as well as the overall period (defined as any assessment for which the included study did not report a specific time point). The secondary outcome was the use of rescue antiemetics. Subgroup analyses were performed according to the timing of administration (preoperative, combined preoperative-and-intraoperative, intraoperative, or postoperative). *Results*: Fourteen RCTs (*n* = 1455), predominantly derived from laparoscopic surgery, were identified as eligible for the systematic review; of these, 13 RCTs (15 sub-studies, 1334 patients) contributed to the quantitative synthesis, while one trial (Firouzian et al.) was included in the qualitative review and risk-of-bias assessment only, as it reported continuous outcome data incompatible with the binary-outcome meta-analysis. Overall, dextrose infusion significantly reduced PON (RR = 0.73; 95% CI: 0.62–0.87), early PON (RR = 0.73; 95% CI: 0.60–0.88), PONV (RR = 0.65; 95% CI: 0.51–0.86), and early PONV (RR = 0.66; 95% CI: 0.51–0.85). Dextrose also significantly reduced the overall use of rescue antiemetics (RR = 0.57; 95% CI: 0.37–0.87). Formal tests for subgroup interaction by administration timing were significant only for rescue antiemetic use (*p* = 0.044); interaction tests for all PON, POV, and PONV outcomes were not statistically significant (all *p* > 0.4), indicating that the timing-based subgroup findings for these outcomes should be considered exploratory. *Conclusions*: Perioperative intravenous dextrose is an effective nonpharmacological adjunct for PONV prevention, with efficacy that may vary according to the timing of administration; however, this timing-based pattern was formally supported by a significant subgroup interaction test only for the use of rescue antiemetics, not for PON, POV, or PONV, and should therefore be regarded as exploratory. In this analysis, postoperative administration was associated with reduced PON and PONV, and preoperative, combined preoperative-and-intraoperative, and postoperative administration were each associated with reduced use of rescue antiemetics, whereas intraoperative administration alone showed no significant benefit.

## 1. Introduction

Postoperative nausea and vomiting (PONV) remains one of the most challenging perioperative complications, and patients frequently rate it as more distressing than the postoperative pain itself [1]. Despite advances in anesthetic and perioperative care, the incidence of PONV in high-risk patients can reach up to 80%, contributing to clinically significant complications such as dehydration, electrolyte imbalance, wound dehiscence, and prolonged hospitalization [2]. These adverse outcomes not only diminish patient satisfaction but also increase healthcare costs owing to greater nursing demands and unplanned readmissions [3].

The pathophysiology of PONV is multifactorial and involves complex interactions between the central and peripheral pathways, including the chemoreceptor trigger zone and vagal afferents. Current pharmacological strategies primarily rely on receptor-specific agents such as 5-HT3 receptor antagonists, corticosteroids, and dopamine antagonists [4]. Emetogenic stimuli arising centrally—such as circulating anesthetic agents and opioids acting on the area postrema, which lacks a complete blood–brain barrier—and peripherally—such as surgical stress-induced release of serotonin from enterochromaffin cells in the gastrointestinal mucosa, activating vagal afferents—converge on the vomiting center in the medullary reticular formation. This integration involves multiple neurotransmitter receptors, including serotonin (5-HT3), dopamine (D2), neurokinin-1 (NK1), histamine (H1), and muscarinic acetylcholine receptors. However, these antiemetic agents are associated with potential adverse effects, including headache, elevated liver enzyme levels, and extrapyramidal symptoms, and their efficacy may be limited when used as monotherapies [5,6].

Additionally, perioperative metabolic status has been suggested as a modifiable factor that may influence the risk of PONV. Preoperative fasting, routinely implemented to reduce the risk of pulmonary aspiration, induces a catabolic state and promotes postoperative insulin resistance [7,8]. In this context, perioperative intravenous dextrose administration (i.e., glucose supplementation) has been proposed as a simple and inexpensive intervention. By supplying an exogenous energy source and attenuating metabolic stress, dextrose infusion has been shown to attenuate perioperative insulin resistance and metabolic stress, which may secondarily influence emetic pathways [9]. However, its clinical efficacy remains unclear.

Several systematic reviews and meta-analyses have evaluated the antiemetic effects of intravenous dextrose administration [10,11,12,13]. However, their findings are inconsistent, and the overall quality of evidence is limited owing to small sample sizes and methodological heterogeneity. More recently, additional large-scale, high-quality randomized controlled trials (RCTs) have been published [14,15,16], providing an opportunity for more robust and comprehensive evaluations.

Therefore, we conducted a systematic review and meta-analysis with trial sequential analysis (TSA) to determine the effect of intravenous dextrose administration on the prevention of PONV.

## 2. Materials and Methods

### 2.1. Protocol Registration and Reporting Guidelines

The study protocol was developed in accordance with the Preferred Reporting Items for Systematic Review and Meta-Analysis Protocols (PRISMA-P) guidelines [17]. The protocol was prospectively registered in the International Prospective Register of Systematic Reviews (PROSPERO; registration number: CRD420261298215) on 2 February 2026. The review was conducted in accordance with the methodological recommendations of the Cochrane Handbook for Systematic Reviews of Interventions, and the findings were reported in accordance with the PRISMA statement [18].

### 2.2. Inclusion and Exclusion Criteria

Predefined eligibility criteria were used to guide the systematic search and the study selection process. RCTs were considered eligible if they evaluated the efficacy and safety of IV dextrose administration for the prevention of PONV in patients undergoing elective surgery compared with controls.

Eligibility was defined according to the PICO-SD framework:

Patients (P): Adult patients undergoing elective surgery under general anesthesia.

Intervention (I): Perioperative intravenous dextrose administration.

Comparison (C): Placebo or intravenous fluids without dextrose.

Outcomes (O):

- Primary outcomes: Incidence of postoperative nausea (PON), postoperative vomiting (POV), and PONV.
- Secondary outcomes: Use of rescue antiemetic medications.

For primary outcomes, the postoperative period was categorized into early, late, and overall phases. The early phase was defined as 0–6 h postoperatively, and the late phase was defined as 6–48 h postoperatively. When studies reported outcomes at multiple time points within the same phase, the earliest time point was selected for analysis (e.g., if data were reported at 0, 2, 4, and 6 h, the 0-h data were used for the early phase) to minimize the inclusion of events accumulating over a longer observation window within the early phase. Any PON, POV, or PONV data from studies that did not mention a specific time point were defined as data from the overall phase, for the same reason: to capture the maximum number of studies without fabricating a time-stratified breakdown the original publication did not provide.

RCTs reported as full-text articles, as well as those reported in abbreviated form such as conference abstracts or correspondence containing original trial data, were eligible.

Case reports, observational studies, comments, letters to the editor, reviews, and animal or laboratory studies were excluded.

### 2.3. Information Source and Search Strategy

A comprehensive and systematic literature search was conducted in PubMed, Ovid-EMBASE, the Cochrane Central Register of Controlled Trials (CENTRAL), and Google Scholar from database inception to 25 February 2026. Two reviewers (JHC and PGP) independently screened the retrieved records to identify eligible studies.

The search strategy incorporated both controlled vocabulary (e.g., MeSH and Emtree terms) and free-text keywords. The main search terms included “postoperative nausea and vomiting,” “nausea,” “vomiting,” “PONV,” “dextrose,” “carbohydrate solution,” “randomized controlled trial,” and “RCT.” The complete, database-specific Boolean search strategies for PubMed, Ovid-EMBASE, CENTRAL, Google Scholar, ClinicalTrials.gov, and OpenSIGLE are provided in the Supplementary Materials (Search Strategy).

In addition, the ClinicalTrials.gov registry was searched to identify completed but unpublished trials, and the OpenSIGLE database was used to retrieve gray literature. The reference lists of all the included studies were manually screened to identify relevant publications. No restrictions were applied regarding language or publication dates. The database and registry search reported above updates and supersedes an earlier, unpublished pilot literature search conducted internally by the same research team, from which ten of the included studies were originally identified; all ten of these studies were re-screened against the eligibility criteria above and underwent new data extraction and risk-of-bias assessment under the present protocol, together with the three additional studies identified through the current search. The ten studies originally identified through the earlier pilot search were also independently retrieved by the current, comprehensive database search, confirming that the updated search strategy was sufficiently sensitive to identify all previously known eligible trials.

### 2.4. Study Selection

Two investigators (JHC and GJC) independently screened all the identified studies based on their titles and abstracts. Studies deemed potentially eligible by either reviewer were retrieved from the full text for further assessment. The full texts were independently evaluated by the same two reviewers according to the predefined inclusion criteria.

Any discrepancies between reviewers were resolved through discussion. If a consensus could not be reached, a third investigator (HK) was consulted to resolve any disagreements.

### 2.5. Data Extraction

Data from the included studies were independently extracted and cross-checked by two investigators (JHC and PGP) using a predefined standardized data collection form. Any discrepancies were resolved through discussion, and if a consensus could not be reached, a third investigator (HK) was consulted.

The following data were extracted: study characteristics (title, first author, journal, publication year, study design, trial registration, funding, and competing interests), study setting (country), and methodological quality (risk of bias). The patient and clinical variables included sample size, sex, age, body weight, height, duration of anesthesia, and American Society of Anesthesiologists (ASA) physical status. The intervention-related variables included the dose and timing of dextrose administration, type of surgery, type of anesthesia, induction and maintenance agents, use of nitrous oxide, perioperative opioid use, and other concomitant medications. Outcome data included definitions of nausea, vomiting, and PONV; the incidence of PON, POV, and PONV across the early, late, and overall phases; and the use of rescue antiemetics.

Quantitative data were extracted from the text, tables, or figures, or calculated when sufficient information was available. When outcomes were reported only graphically, numerical values were estimated using the Plot Digitizer software (version 2.6.8; http://plotdigitizer.sourceforge.net, accessed on 1 May 2026). For missing or incomplete data, the corresponding authors were contacted to obtain additional information.

### 2.6. Data Analysis

#### 2.6.1. Conventional Meta-Analysis

The meta-analysis was performed using R (version 4.5.3; R Foundation for Statistical Computing, Vienna, Austria), employing the meta and metafor packages. The data were independently entered by two investigators (JHC and HK) and cross-checked for accuracy. For each outcome, pooled risk ratios (RRs) with 95% confidence intervals (CIs) were calculated using the metabin function. In trials reporting more than one comparison (e.g., multiple dextrose dosing arms versus a single control arm), each comparison was treated as an independent sub-study in the meta-analysis. When a shared control arm was used across multiple comparisons within a trial, the control group sample size and event count were divided equally among the comparisons to avoid double-counting. For sub-study comparisons with zero events in both arms (double-zero), a continuity correction of 0.1 was added to each cell prior to pooling. Comparisons with zero events in only one arm (single-zero) were incorporated into the exact Mantel–Haenszel pooled estimate without a continuity correction, consistent with the properties of the exact method for handling single-zero cells. Between-study heterogeneity was assessed using the chi-squared (Q) test, Higgins’ I^2^ statistic, and τ^2^ estimated with the Paule–Mandel method. In addition, 95% prediction intervals (PrIs) were calculated to assess the dispersion of true effects across studies [19]. Heterogeneity was considered substantial when I^2^ exceeded 50% or when the Q test yielded a *p*-value < 0.10. Given the anticipated clinical and methodological heterogeneity, a random-effects model was used for all pooled analyses, with 95% confidence intervals calculated using the classic (Wald-type) method. As a sensitivity analysis, confidence intervals were additionally recalculated using the Hartung–Knapp adjustment, which accounts for the uncertainty in the between-study variance estimate and is generally recommended when the number of pooled studies is small. As a further sensitivity analysis, τ^2^ was re-estimated using restricted maximum likelihood (REML), combined with the Hartung–Knapp adjustment, and compared with the Paule–Mandel-based results. The robustness of the pooled estimates was further evaluated using leave-one-out sensitivity analyses. As an additional sensitivity analysis, studies contributing to the “overall” phase from a single, unstratified or approximate time point rather than a study-defined overall assessment were excluded from the Overall PON, POV, and PONV syntheses to assess the robustness of these estimates. A prespecified subgroup analysis was conducted according to the timing of dextrose administration: preoperative, both preoperative and intraoperative, intraoperative, and postoperative. For each outcome, a formal test for subgroup interaction was performed by comparing the random-effects estimates across timing subgroups, each with a separately estimated between-study variance, using a chi-squared test with degrees of freedom equal to the number of subgroups minus one. Post hoc subgroup analyses by surgery type (laparoscopic vs. non-laparoscopic) and a random-effects meta-regression of log(RR) against mean surgical duration were also performed for the overall PON, POV, and PONV outcomes to explore additional potential sources of heterogeneity. A sensitivity analysis by specific anesthetic agent was planned.

Publication bias was assessed only for outcomes including ≥ 10 substudies. Contour-enhanced funnel plots and Egger’s regression tests were used to assess for publication bias. The Duval and Tukey trim-and-fill method was applied to estimate the number of potentially missing studies and to provide a bias-adjusted pooled estimate. Sensitivity analyses for potential publication bias were further conducted using: (1) Vevea–Hedges selection models with three prespecified scenarios assuming moderate (*p* < 0.05 reported 2-fold more often), strong (3-fold), and gradient selection (*p* < 0.01: 2-fold, 0.01 ≤ *p* < 0.05: 1.5-fold); (2) PET-PEESE regression-based estimators; and (3) Bayesian analysis using a normal–normal conjugate update with weakly informative (precision = 1), skeptical (precision = 5), and very skeptical (precision = 10) priors centered at the null effect, combined with the same three selection scenarios [20].

The number needed to treat (NNT) was calculated with corresponding 95% CIs based on the absolute risk reduction to quantify the clinical relevance of the intervention [21].

#### 2.6.2. Trial Sequential Analysis

Trial sequential analysis (TSA) was performed to estimate the required information size (RIS) and determine whether the available evidence was sufficient to draw firm conclusions [22]. A random-effects model based on the DerSimonian–Laird method was used to construct the cumulative Z-curve. TSA was conducted with an overall type I error rate of 5% and type II error of 20%.

When the cumulative Z-curve crossed the monitoring boundary or entered the futility boundary, we considered the evidence to be sufficient to confirm or rule out the expected effects of the intervention, indicating that additional trials were probably unnecessary. Conversely, if the Z-curve did not cross any boundary and RIS was not reached, the evidence was considered inconclusive, indicating the need for additional studies.

The RIS was calculated based on the observed event proportion in the control group (i.e., the cumulative proportion of participants with events across control groups), assuming a relative risk reduction of 20% in the dextrose group, a type I error (α) of 5%, a type II error (β) of 20% (power of 80%), and the diversity (D^2^) estimated from the included trials. All trial sequential analyses were performed using the TSA software (Copenhagen Trial Unit), version 0.9.5.10, with studies ordered chronologically by year of publication. The control event proportion, D^2^, and RIS used for each individual outcome are reported in Supplementary Table S1.

#### 2.6.3. Risk of Bias Assessment

Two investigators (JHC and GJC) independently assessed the methodological quality of the included studies using the revised Cochrane risk-of-bias tool for randomized trials (RoB 2.0) [23]. The following domains were evaluated for each study: (1) bias arising from the randomization process; (2) bias due to deviations from the intended interventions; (3) bias due to missing outcome data; (4) bias in outcome measurement; and (5) bias in the selection of the reported results.

An overall risk of bias judgment was assigned according to the RoB 2.0 guidance: “low risk” when all domains were rated as low risk; “high risk” when at least one domain was rated as high risk or multiple domains raised some concerns; and “some concerns” when the overall assessment did not meet the criteria for either low or high risk.

Discrepancies between the reviewers were resolved through discussion; if a consensus could not be reached, a third investigator (HK) was consulted.

#### 2.6.4. Quality of Evidence

The certainty of evidence for each outcome was assessed using the Grading of Recommendations, Assessment, Development, and Evaluation (GRADE) approach [24]. This framework considers the overall quality of evidence, the balance between benefits and harms, and the strength of recommendations.

The certainty of evidence was categorized into four levels: high, indicating that further research is unlikely to change confidence in the effect estimate; moderate, indicating that further research is likely to have an important impact on confidence in the estimate and may change the estimate; low, indicating that further research is likely to have an important impact on confidence in the estimate and is likely to change the estimate; and very low, indicating that the effect estimate is highly uncertain.

## 3. Results

### 3.1. Literature Search and Study Selection

The database and register searches yielded 454 records (434 from the databases and 20 from the trial registries), of which 33 duplicates were excluded prior to screening. The remaining 421 records were screened by title and abstract, of which 412 were excluded because they were irrelevant. Nine reports were retrieved, and all were obtained for full-text assessment. Of these, six were excluded for the following reasons: narrative review (*n* = 1) [25], letter [26], not reporting the outcome of interest (*n* = 1) [27], not an RCT (*n* = 1) [28], performed in patients under sedation (*n* = 1) [29], and oral carbohydrate intake rather than intravenous dextrose (*n* = 1) [30].

**Figure 1:**
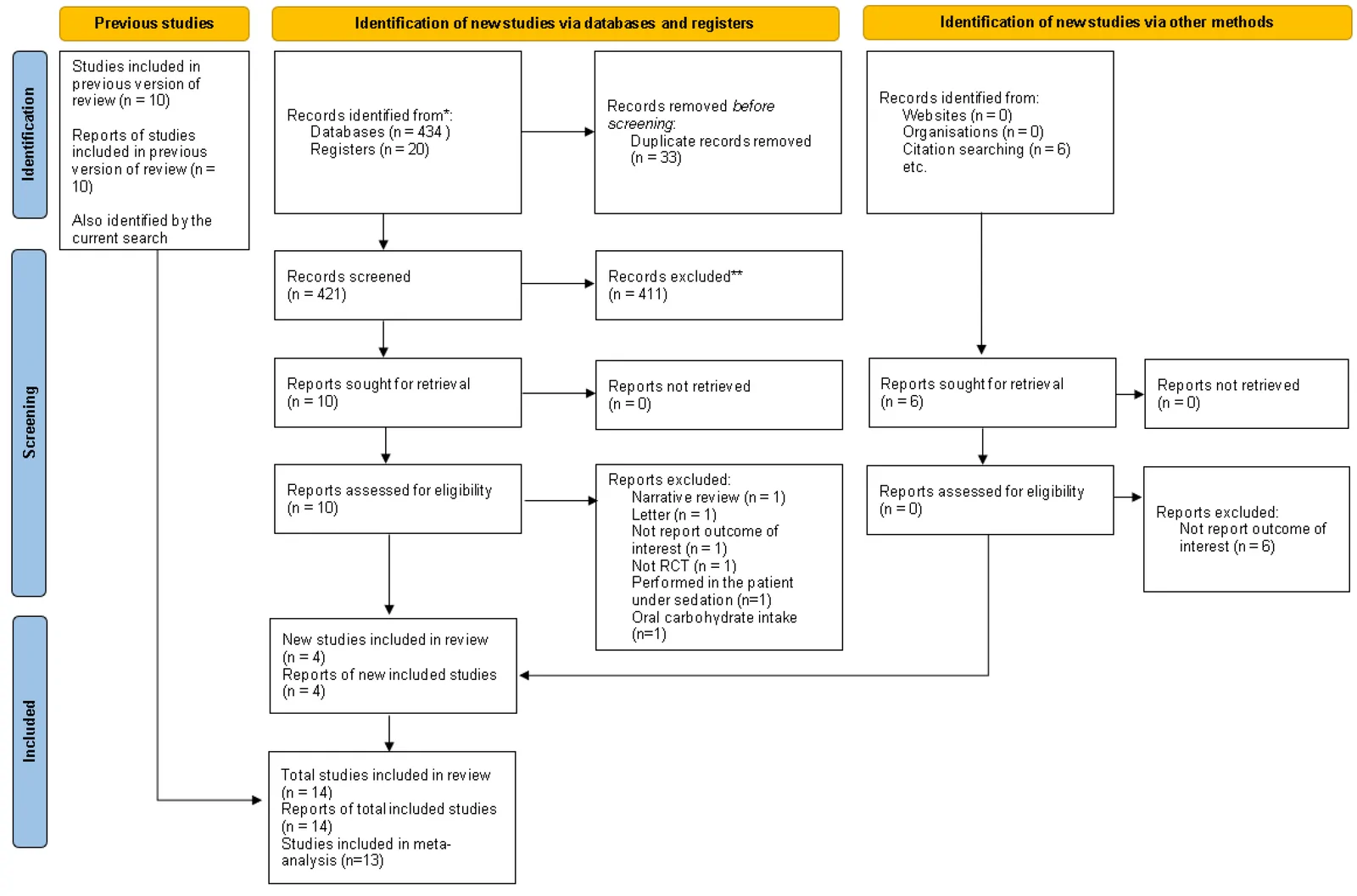
PRISMA 2020 flow diagram showing the identification, screening, and inclusion of studies for this updated systematic review and meta-analysis. * Records identified from PubMed, Ovid-EMBASE, CENTRAL, Google Scholar, ClinicalTrials.gov, and OpenSIGLE. ** Records excluded during title and abstract screening because they were considered irrelevant to the review question.

Therefore, three new studies were added through database and register searches. The OpenSIGLE grey-literature search did not identify any additional eligible records. An additional six records were identified through a citation search; all six were excluded after a full-text review because they did not report the outcome of interest. Combined with the ten studies retained from the earlier pilot search—all of which were also independently retrieved by the current comprehensive search—14 studies were included in the systematic review, of which 13 contributed to the updated meta-analysis (Figure 1).

### 3.2. Study Characteristics

A total of 14 randomized controlled trials (*n* = 1455) were identified as eligible for the systematic review (Table 1). Of these, 13 trials (*n* = 1385), comprising 15 sub-studies, contributed to the quantitative meta-analysis; Firouzian et al. was included in the qualitative synthesis and risk-of-bias assessment only, as it reported continuous outcome data incompatible with the binary-outcome meta-analysis (Table 1).

All studies were performed under general anesthesia. The types of surgery included laparoscopy [31], gynecologic laparoscopy [16,32,34,39,40], gynecologic, plastic, and breast surgery [33], middle-ear surgery [35], laparoscopic cholecystectomy [15,36,37,41], and laparoscopic sleeve gastrectomy [14]. Of the 13 included trials, 11 (85%) involved laparoscopic procedures (laparoscopy, gynecologic laparoscopy, laparoscopic cholecystectomy, or laparoscopic sleeve gastrectomy), while two involved non-laparoscopic surgery (gynecologic/plastic/breast surgery and middle-ear surgery). The mean reported surgical duration across the included trials was approximately 86 min (range, 20–173 min; individual study durations are listed in Table 1). Because the evidence base is predominantly derived from laparoscopic surgery, the findings of this review should be interpreted as most directly applicable to that setting.

The intravenous dextrose solutions varied across studies: 5% dextrose in LRS [16,31,33,34,35,36,37,39], 5% dextrose in CSL [32], 5% dextrose in NSS [40], 5% dextrose in 0.45% saline [41], 5% dextrose [15], and 0.9% sodium chloride combined with 5% dextrose [14].

The timing of dextrose administration was classified into four categories: preoperative only [15,41], both preoperative and intraoperative [15,39], intraoperative only [14,31,32,33,36,40,41] and postoperative [16,34,35,37].

### 3.3. PON (Overall Phase, Early and Late)

**Figure 2:**
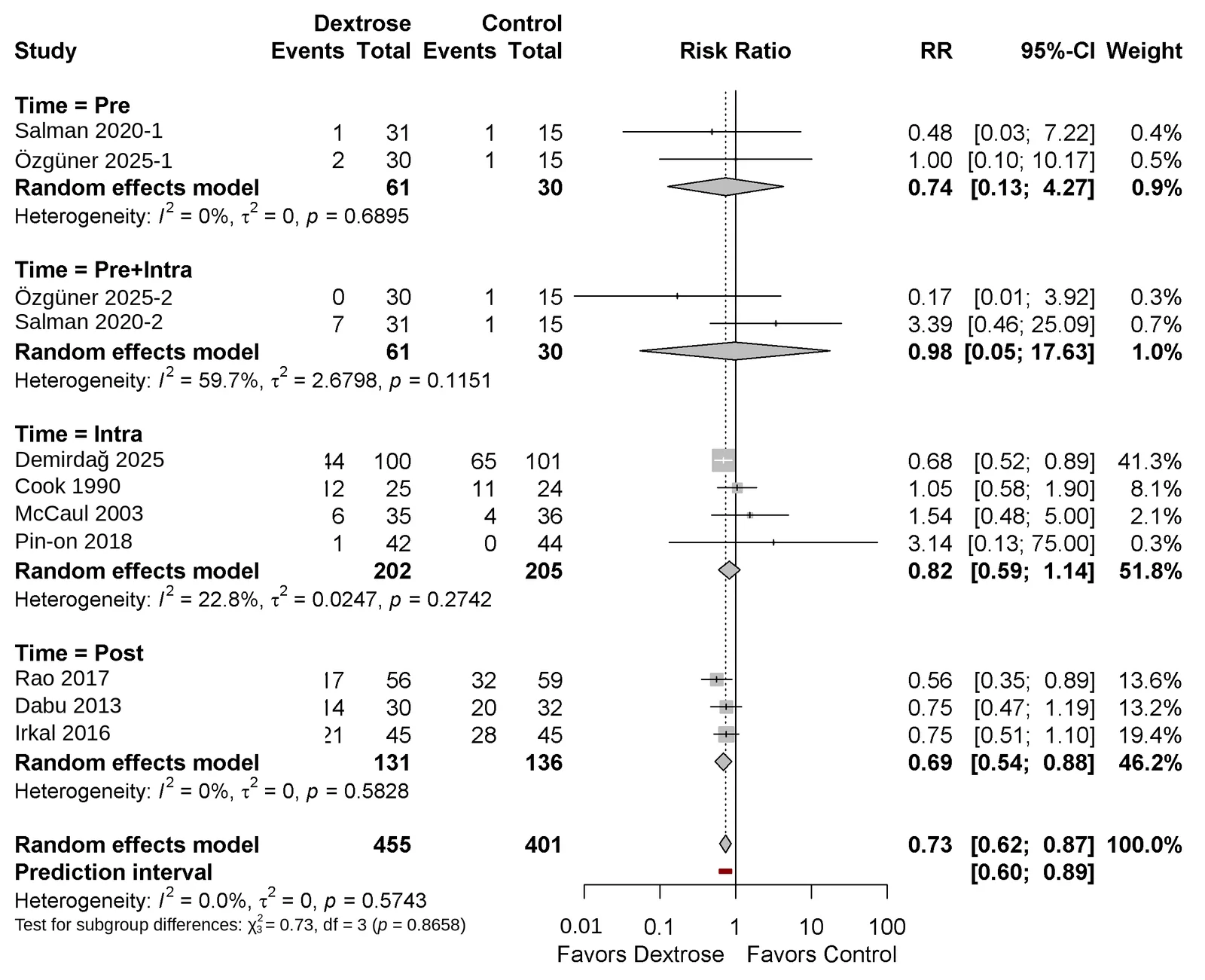
Forest plot for the incidence of overall postoperative nausea (PON) stratified by the timing of dextrose administration. Each study is represented by a filled square proportional to its weight, with horizontal lines indicating the 95% confidence interval (CI). The black diamonds represent the subgroup-specific pooled risk ratios (RRs), and the open diamond indicates the overall pooled RR for all studies. Vertical dashed lines represent the null effect (RR = 1) [14,15,31,32,34,35,37,40,41].

Nine studies involving 856 patients reported the overall incidence of PON [14,15,31,32,34,35,37,40,41]. The incidence of overall PON was significantly lower in the dextrose group (27.4%; 125/455 patients) than in the control group (40.8%; 164/402 patients) (RR = 0.73, 95% CI: 0.62–0.87, I^2^ = 0.0%, P_chi_^2^ = 0.574, τ^2^ = 0.0, 95% PrI: 0.60–0.89, NNTB = 7.4, 95% CI: 5.1–14.1) (Figure 2).

Subgroup analysis according to the timing of dextrose administration showed no significant difference between groups when dextrose was administered preoperatively (RR = 0.74, 95% CI: 0.13–4.27, I^2^ = 0.0, P_chi_^2^ = 0.690, τ^2^ = 0.0), both preoperatively and intraoperatively (RR = 0.98, 95% CI: 0.05–17.63, I^2^ = 59.7, P_chi_^2^ = 0.115, τ^2^ = 2.680), or intraoperatively alone (RR = 0.82, 95% CI: 0.59–1.14, I^2^ = 22.8, P_chi_^2^ = 0.274, τ^2^ = 0.025). However, when dextrose was administered postoperatively, the incidence of overall PON was significantly lower in the dextrose group than in the control group (RR = 0.69, 95% CI: 0.54–0.88, I^2^ = 0.0, P_chi_^2^ = 0.583, τ^2^ = 0.0) (Figure 2).

Sensitivity analysis using a leave-one-out approach showed that the removal of a single study did not change the statistical significance of the pooled results (Supplementary Figure S1).

**Figure 3:**
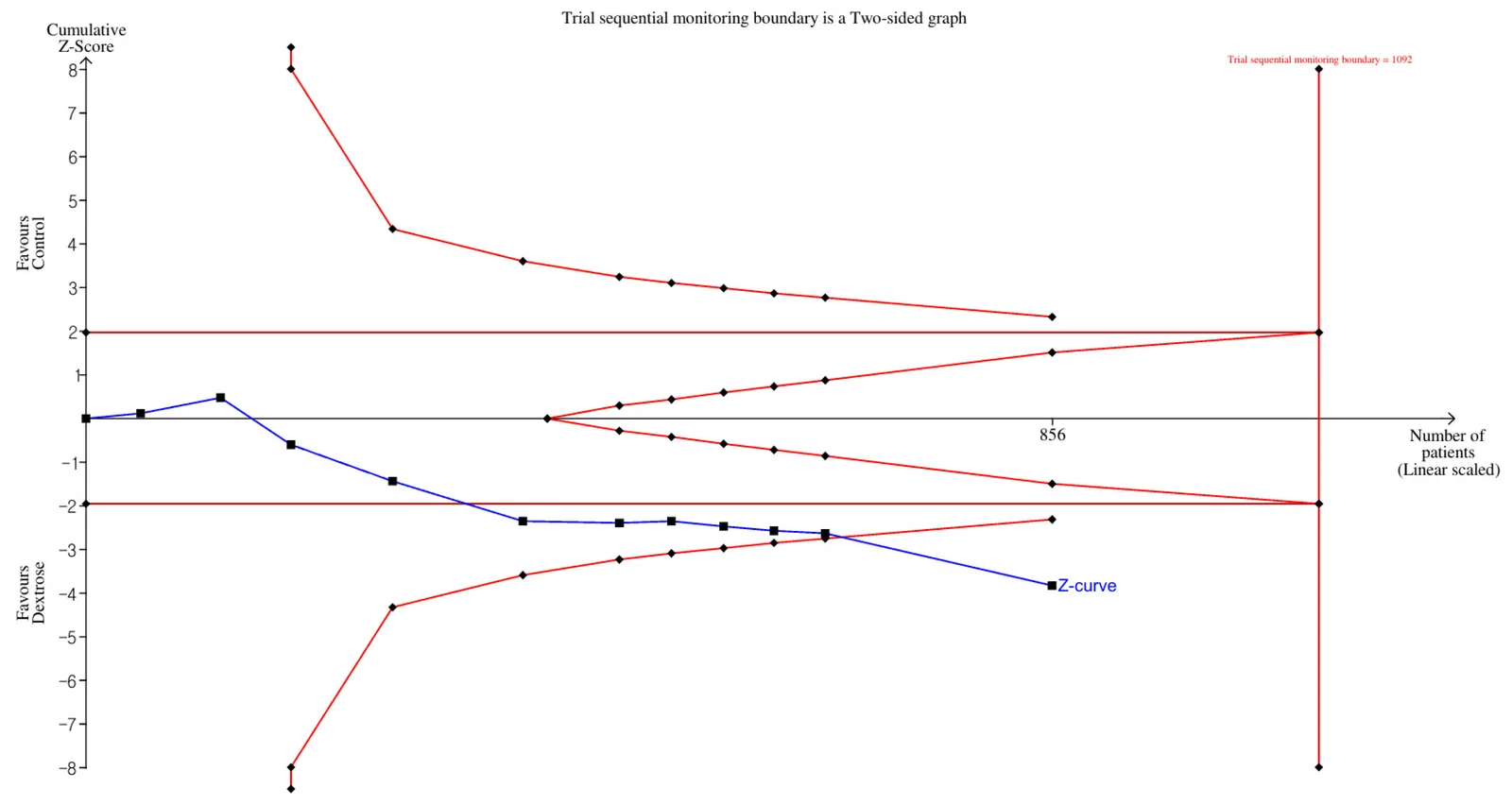
Trial sequential analysis (TSA) plot for the incidence of overall postoperative nausea (PON). The uppermost and lowermost continuous red lines represent the trial sequential monitoring boundaries for benefit and harm, respectively. The horizontal red lines indicate the conventional boundaries for statistical significance. The triangular red lines extending to the right represent the futility boundaries. The blue solid line represents the cumulative Z-curve, which crosses the trial sequential monitoring boundary, indicating that the finding remained statistically significant after adjustment for the risk of Type I error associated with repeated testing. This finding should be interpreted alongside the conventional meta-analysis, sensitivity analyses, and the corresponding GRADE certainty rating, rather than being regarded as definitive evidence that no further trials are needed. The vertical red line on the far right indicates the required information size (RIS) of 1092 patients.

TSA indicated that 78.4% (856 of 1092 patients) of the RIS was accrued. The cumulative Z-curve crossed both the conventional test boundary and the trial sequential monitoring boundary (Figure 3).

Eight studies (*n* = 766) reported the incidence of early PON [14,15,31,32,34,37,40,41]. The incidence of early PON was significantly lower (RR = 0.73, 95% CI: 0.60 to 0.88), I^2^ = 0.0, P_chi_^2^ = 0.481, τ^2^ = 0.0, 95% PrI: 0.58 to 0.90, NNTB = 7.8, 95% CI = NNTB 5.2 to NNTB 16.0) in the dextrose (25.3%; 104 of 410 patients) group than in the control group (35.0%; 136 of 356 patients) (Supplementary Figure S2).

Subgroup analysis according to the timing of dextrose administration showed no significant difference between groups when dextrose was administered preoperatively (RR = 0.74, 95% CI: 0.13–4.27, I^2^ = 0.0, P_chi_^2^ = 0.690, τ^2^ = 0.0), both preoperatively and intraoperatively (RR = 0.98, 95% CI: 0.05–17.63, I^2^ = 59.7, P_chi_^2^ = 0.115, τ^2^ = 2.680), or intraoperatively alone (RR = 0.82, 95% CI: 0.59–1.14, I^2^ = 22.8, P_chi_^2^ = 0.274, τ^2^ = 0.025). However, when dextrose was administered postoperatively, the incidence of early PON was significantly lower in the dextrose group than in the control group (RR = 0.65, 95% CI: 0.46 to 0.90, I^2^ = 0.0, P_chi_^2^ = 0.389, τ^2^ = 0.0) (Supplementary Figure S2).

Sensitivity analyses, removal of a single study, yielded a change in statistical significance when the Demirdağ et al. study [14] was removed (Supplementary Figure S3).

TSA indicated that only 63.1% (766 of 1214 patients) of the RIS was accrued. The cumulative Z-curve crossed both the conventional test boundary and the trial sequential monitoring boundary. (Supplementary Figure S4).

Six studies (633 patients) reported the incidence of late PON [14,15,31,37,40,41]. The combined results showed no evidence of a difference (RR = 0.83, 95% CI: 0.54 to 1.28, I^2^ = 11.8, P_chi_^2^ = 0.338, τ^2^ = 0.038, 95% PrI: 0.42 to 1.67, NNTB = 24.1, 95% CI = NNTH 62.2 to ∞ to NNTB 10.1) between the control (18.1%; 52 of 288 patients) and dextrose (13.9%; 48 of 345 patients) groups (Supplementary Figure S5).

Subgroup analysis according to the timing of dextrose administration showed no significant difference between groups when dextrose was administered preoperatively (RR = 0.35, 95% CI: 0.03 to 4.49, I^2^ = 59.7, P_chi_^2^ = 0.115, τ^2^ = 2.105), both preoperatively and intraoperatively (RR = 0.41, 95% CI: 0.09 to 1.78, I^2^ = 16.7, P_chi_^2^ = 0.273, τ^2^ = 0.197), intraoperatively alone (RR = 1.10, 95% CI: 0.73 to 1.66, I^2^ = 0.0, P_chi_^2^ = 0.567, τ^2^ = 0.0), or postoperatively (RR = 0.67, 95% CI: 0.28 to 1.61) (Supplementary Figure S5).

Sensitivity analyses and removal of a single study at a time did not change the statistical significance (Supplementary Figure S6).

TSA indicated that only 10.9% (633 of 5784 patients) of the RIS was accrued. The cumulative Z-curve did not cross the conventional test boundary (Supplementary Figure S7).

### 3.4. POV (Overall Phase)

To maximize data inclusion in the overall phase, outcomes (PON, POV, and PONV) from studies without clearly defined time points were classified as overall. Consequently, for POV, the counts in the overall and early phases are identical.

Nine studies (856 patients) reported the overall incidence of POV [14,15,31,32,34,35,37,40,41]. The combined results showed no evidence of a difference (RR = 0.84, 95% CI = 0.52 to 1.38, I^2^ = 0.0, P_chi_^2^ = 0.940, τ^2^ = 0.0, 95% PrI: 0.48 to 1.47, NNTB = 64.6, 95% CI = NNTH 54.9 to ∞ to NNTB 20.3) between the control (7.5%; 30 of 401 patients) and dextrose (5.9%; 27 of 455 patients) groups (Supplementary Figure S8).

Subgroup analysis according to the timing of dextrose administration showed no significant difference between groups when dextrose was administered preoperatively (RR = 0.68, 95% CI = 0.05 to 9.32, I^2^ = 31.9, P_chi_^2^ = 0.226, τ^2^ = 1.143), both preoperatively and intraoperatively (RR = 0.79, 95% CI = 0.10 to 6.14, I^2^ = 0.0, P_chi_^2^ = 0.609, τ^2^ = 0.0), intraoperatively alone (RR = 1.17, 95% CI = 0.46 to 2.99, I^2^ = 0.0, P_chi_^2^ = 0.872, τ^2^ = 0.0), or postoperatively (RR = 0.74, 95% CI = 0.40 to 1.39, I^2^ = 0.0, P_chi_^2^ = 0.591, τ^2^ = 0.0) (Supplementary Figure S8).

Sensitivity analyses and removal of a single study at a time did not change the statistical significance (Supplementary Figure S9).

TSA indicated that only 9.7% (856 of 8783 patients) of the RIS was accrued. The cumulative Z-curve did not cross the conventional test boundary (Supplementary Figure S10).

Six studies (633 patients) reported on the incidence of late POV [14,15,31,37,40,41]. The combined results showed no evidence of a difference (RR = 1.09, 95% CI = 0.48 to 2.47, I^2^ = 0.0, P_chi_^2^ = 0.986, τ^2^ = 0.0, 95% PrI: 0.40 to 2.93, NNTH = 338.0, 95% CI = NNTH 31.1 to ∞ to NNTB 38.2) in the control (3.5%; 10 of 288 patients) and dextrose (3.7%; 13 of 345 patients) groups (Supplementary Figure S11).

Subgroup analysis according to the timing of dextrose administration showed no significant difference between groups when dextrose was administered preoperatively (RR = 2.19, 95% CI = 0.01 to 397.16, I^2^ = 0.0, P_chi_^2^ = 0.674, τ^2^ = 0.0), both preoperatively and intraoperatively (RR = 0.49, 95% CI = 0.00 to 238.17, I^2^ = 0.0, P_chi_^2^ = 0.996, τ^2^ = 0.0), intraoperatively alone (RR = 1.23, 95% CI = 0.47 to 3.21, I^2^ = 0.0, P_chi_^2^ = 0.674, τ^2^ = 0.0), or postoperatively (RR = 0.70, 95% CI = 0.12 to 4.05) (Supplementary Figure S11).

Sensitivity analyses and removal of this single study at a time did not change the statistical significance (Supplementary Figure S12).

TSA indicated that only 3.2% (633 of 19,548 patients) of the RIS was accrued. The cumulative Z-curve did not cross the conventional test boundary (Supplementary Figure S13).

### 3.5. PONV (Overall Phase, Early and Late)

**Figure 4:**
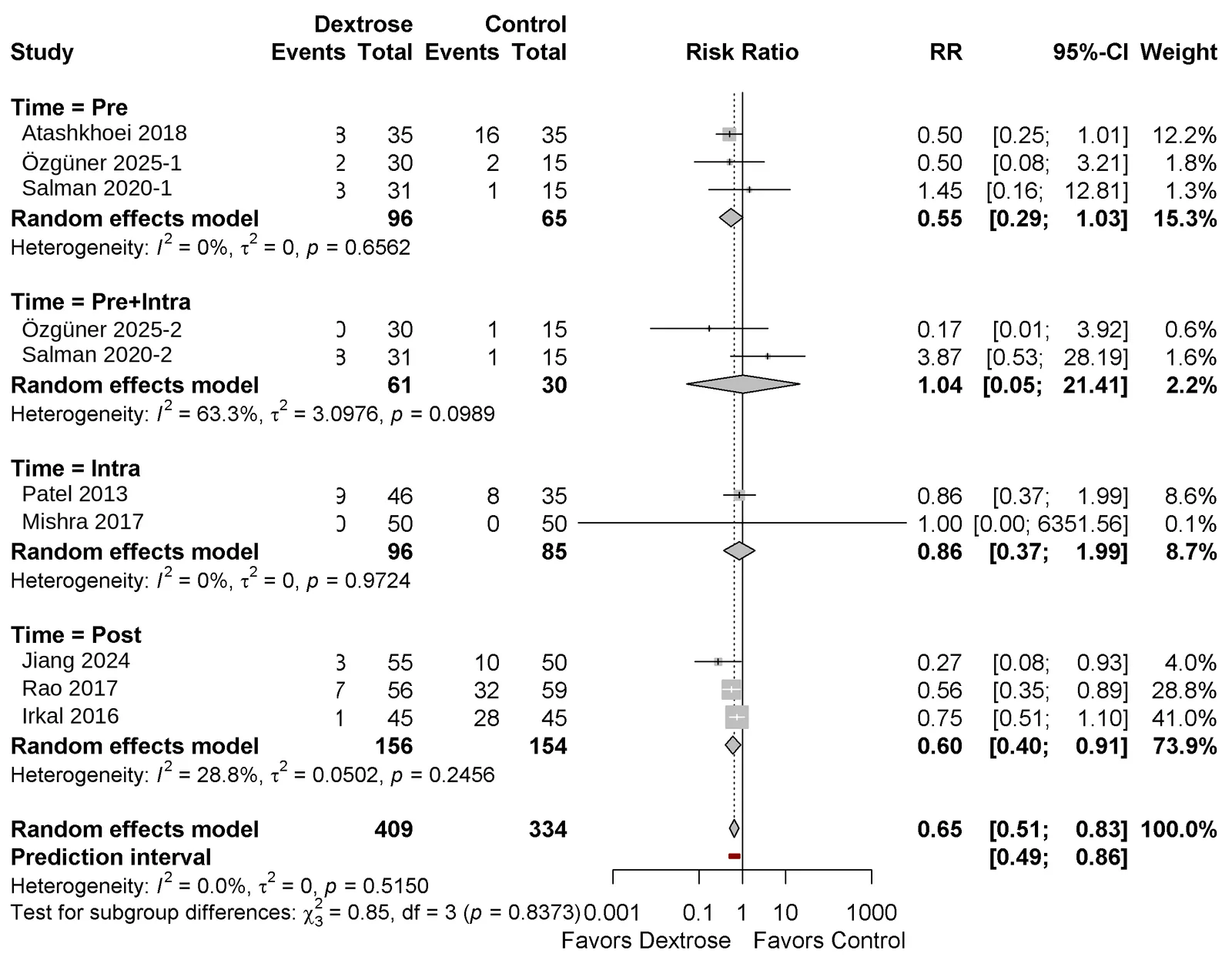
Forest plot for the incidence of overall postoperative nausea and vomiting (PONV) stratified by the timing of dextrose administration. Each study is represented by a filled square proportional to its weight, with horizontal lines indicating the 95% confidence interval (CI). The black diamonds represent the subgroup-specific pooled risk ratios (RRs), and the open diamond at the bottom indicates the overall pooled RR for all included studies. The red horizontal bar represents the prediction interval. Vertical dashed lines represent the null effect (RR = 1) [15,29,33,35,36,37,39,41].

Eight studies (743 patients) reported the overall incidence of PONV [15,16,31,33,35,36,37,39]. The incidence of overall PONV was significantly lower (RR = 0.65, 95% CI: 0.51 to 0.86, I^2^ = 0.0, P_chi_^2^ = 0.515, τ^2^ = 0.0, 95% PrI: 0.49 to 0.86, NNTB = 8.1, 95% CI = NNTB 5.4 to NNTB 16.2) in the dextrose (17.4%; 71 of 409 patients) group than in the control group (29.6%; 99 of 334 patients) (Figure 4).

Subgroup analysis according to the timing of dextrose administration showed no significant difference between groups when dextrose was administered preoperatively (RR = 0.55, 95% CI = 0.29 to 1.03, I^2^ = 0.0, P_chi_^2^ = 0.656, τ^2^ = 0.0), both preoperatively and intraoperatively (RR = 1.04, 95% CI = 0.05 to 21.41, I^2^ = 63.3, P_chi_^2^ = 0.099, τ^2^ = 3.098), or intraoperatively alone (RR = 0.86, 95% CI = 0.37 to 1.99, I^2^ = 0.0, P_chi_^2^ = 0.972, τ^2^ = 0.0). However, when dextrose was administered postoperatively, the incidence of overall PON was significantly lower in the dextrose group than in the control group (RR = 0.60, 95% CI = 0.40 to 0.91, I^2^ = 28.8, P_chi_^2^ = 0.246, τ^2^ = 0.050) (Figure 4).

Sensitivity analyses and removal of this single study at a time did not change the statistical significance (Supplementary Figure S14).

**Figure 5:**
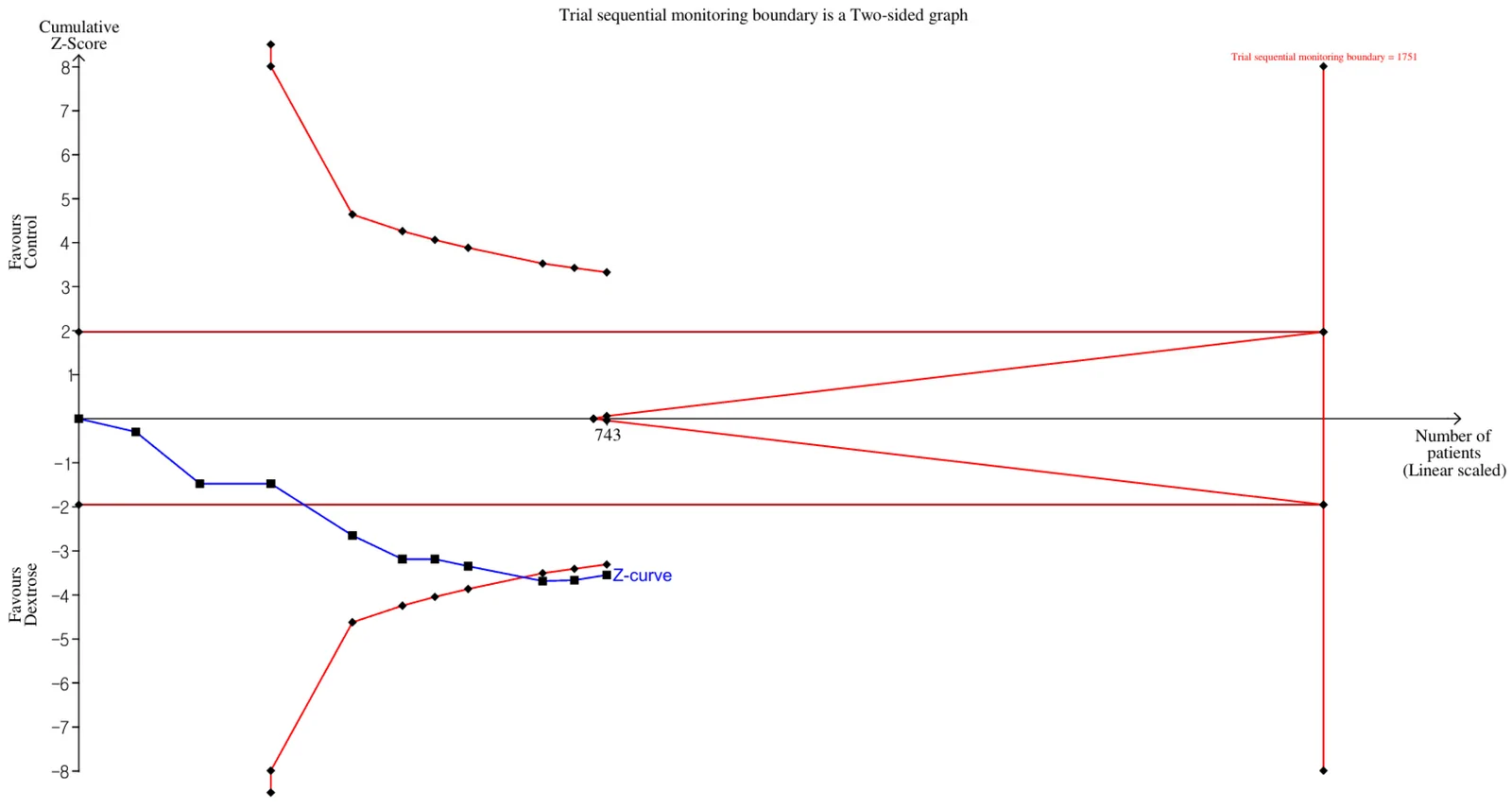
Trial sequential analysis (TSA) plot for the incidence of overall postoperative nausea and vomiting (PONV). The uppermost and lowermost continuous red lines represent the trial sequential monitoring boundaries for benefit and harm, respectively. The horizontal red lines indicate the conventional boundaries for statistical significance. The triangular red lines extending to the right represent the futility boundaries. The blue solid line represents the cumulative Z-curve, which crosses the trial sequential monitoring boundary, indicating that the finding remained statistically significant after adjustment for the risk of Type I error associated with repeated testing. This finding should be interpreted alongside the conventional meta-analysis, sensitivity analyses, and the corresponding GRADE certainty rating, rather than being regarded as definitive evidence that no further trials are needed. The vertical red line on the far right indicates the required information size (RIS) of 1751 patients.

TSA indicated that only 42.4% (743 of 1751 patients) of the RIS was accrued. The cumulative Z-curve crossed both the conventional test boundary and the trial sequential monitoring boundary (Figure 5).

Eight studies (743 patients) reported the incidence of early PONV [15,16,31,33,35,36,37,39]. The incidence of early PONV was significantly lower (RR = 0.66, 95% CI = 0.51 to 0.85, I^2^ = 0.0, P_chi_^2^ = 0.521, τ^2^ = 0.0, 95% PrI: 0.49 to 0.88, NNTB = 9.0, 95% CI = NNTB 5.8 to NNTB 19.3) in the dextrose (16.4%; 67 of 409 patients) group than in the control group (27.5%; 92 of 334 patients) (Supplementary Figure S15).

Subgroup analysis according to the timing of dextrose administration showed no significant difference between groups when dextrose was administered preoperatively (RR = 0.55, 95% CI = 0.12 to 1.29, I^2^ = 0.0, P_chi_^2^ = 0.631, τ^2^ = 0.0), both preoperatively and intraoperatively (RR = 1.04, 95% CI = 0.05 to 21.41, I^2^ = 63.3, P_chi_^2^ = 0.099, τ^2^ = 3.098), or intraoperatively alone (RR = 0.86, 95% CI = 0.37 to 1.99, I^2^ = 0.0, P_chi_^2^ = 0.972, τ^2^ = 0.0). However, when dextrose was administered postoperatively, the incidence of early PONV was significantly lower in the dextrose group than in the control group (RR = 0.60, 95% CI = 0.40 to 0.91, I^2^ = 28.8, P_chi_^2^ = 0.246, τ^2^ = 0.050) (Supplementary Figure S15).

Sensitivity analyses and removal of this single study at a time did not change the statistical significance (Supplementary Figure S16).

TSA indicated that only 38.4% (743 of 1933 patients) of the RIS was accrued. The cumulative Z-curve crossed the conventional test boundary, but did not cross the trial sequential monitoring boundary (Supplementary Figure S17).

Six studies (*n* = 572) reported the incidence of late PONV [15,16,36,37,39,41]. The incidence of late PONV was significantly lower (RR = 0.56, 95% CI = 0.36 to 0.85, I^2^ = 0.0, P_chi_^2^ = 0.734, τ^2^ = 0.0, 95% PrI: 0.33 to 0.93, NNTB = 11.7, 95% CI = NNTB 7.1 to NNTB 34.3) in the dextrose (8.8%; 28 of 318 patients) group than in the control group (17.3%; 44 of 254 patients) (Supplementary Figure S18).

Subgroup analysis according to the timing of dextrose administration showed no significant difference between groups when dextrose was administered preoperatively (RR = 0.39, 95% CI = 0.10 to 1.59, I^2^ = 26.3, P_chi_^2^ = 0.257, τ^2^ = 0.391), both preoperatively and intraoperatively (RR = 0.35, 95% CI = 0.10 to 1.19, ^2^ = 0.0, P_chi_^2^ = 0.399, τ^2^ = 0.0), intraoperatively alone (RR = 0.33, 95% CI = 0.01 to 7.99), or postoperatively (RR = 0.63, 95% CI = 0.38 to 1.04, I^2^ = 0.0, P_chi_^2^ = 0.734, τ^2^ = 0.0) (Supplementary Figure S18).

Sensitivity analyses, that is, the removal of a single study, yielded a change in statistical significance when the study by Liu et al. [16] was excluded (Supplementary Figure S19).

TSA indicated that only 16.6% (572 of 3448 patients) of the RIS was accrued. The cumulative Z-curve crossed the conventional test boundary, but did not cross the trial sequential monitoring boundary (Supplementary Figure S20).

### 3.6. Use of Rescue Antiemetics

**Figure 6:**
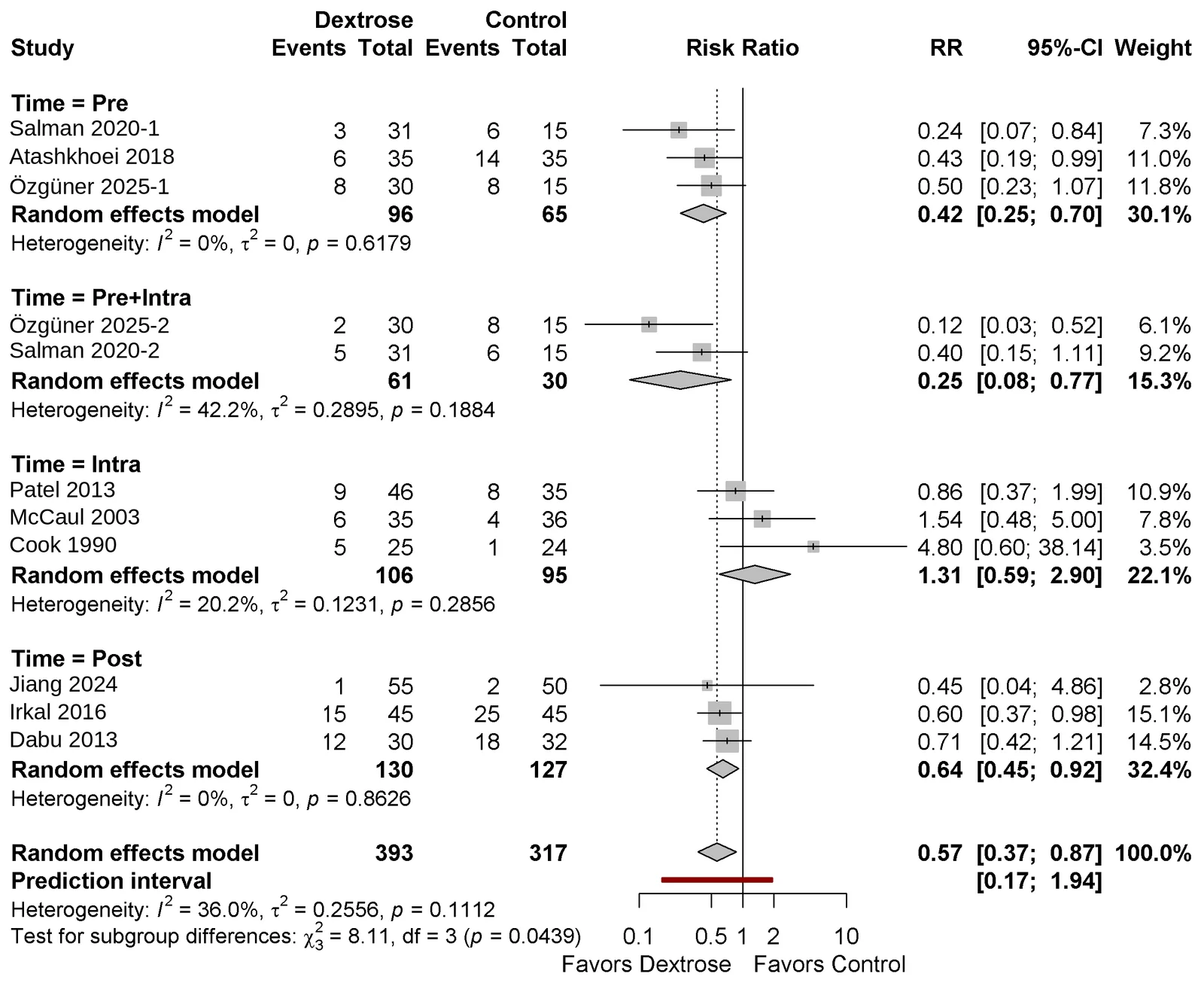
Forest plot for the incidence of use of rescue antiemetics. Each study is represented by a filled square proportional to its weight, with horizontal lines indicating the 95% confidence interval (CI). The black diamonds represent the subgroup-specific pooled risk ratios (RRs), and the open diamond at the bottom indicates the overall pooled RR for all included studies. The red horizontal bar represents the prediction interval. Vertical dashed lines represent the null effect (RR = 1) [15,29,31,32,33,34,35,39,41].

Nine studies (710 patients) reported the incidence of rescue antiemetics [15,16,31,32,33,34,35,39,41]. The incidence of use of rescue antiemetics was significantly lower (RR = 0.57, 95% CI: 0.37 to 0.87, I^2^ = 36.0, P_chi_^2^ = 0.111, τ^2^ = 0.256, 95% PrI: 0.17 to 1.94, NNTB = 7.6, 95% CI = NNTB 5.1 to NNTB 14.6) in the dextrose (18.3%; 72 of 393 patients) group than in the control group (31.5%; 100 of 317 patients) (Figure 6).

Subgroup analysis according to the timing of dextrose administration showed no significant difference between groups when dextrose was administered intraoperatively alone (RR = 1.31, 95% CI = 0.59 to 2.90, I^2^ = 20.2, P_chi_^2^ = 0.286, τ^2^ = 0.123). However, the incidence of use of rescue antiemetics was significantly lower in the dextrose group than in the control group when dextrose was administered preoperatively (RR = 0.42, 95% CI = 0.25 to 0.70, I^2^ = 0.0, P_chi_^2^ = 0.618, τ^2^ = 0.0), both preoperatively and intraoperatively (RR = 0.25, 95% CI = 0.08 to 0.77, I^2^ = 42.2, P_chi_^2^ = 0.188, τ^2^ = 0.289), or postoperatively (RR = 0.64, 95% CI = 0.45 to 0.92, I^2^ = 0.0, P_chi_^2^ = 0.863, τ^2^ = 0.0) (Figure 6).

Sensitivity analyses and removal of this single study at a time did not change the statistical significance (Supplementary Figure S21).

**Figure 7:**
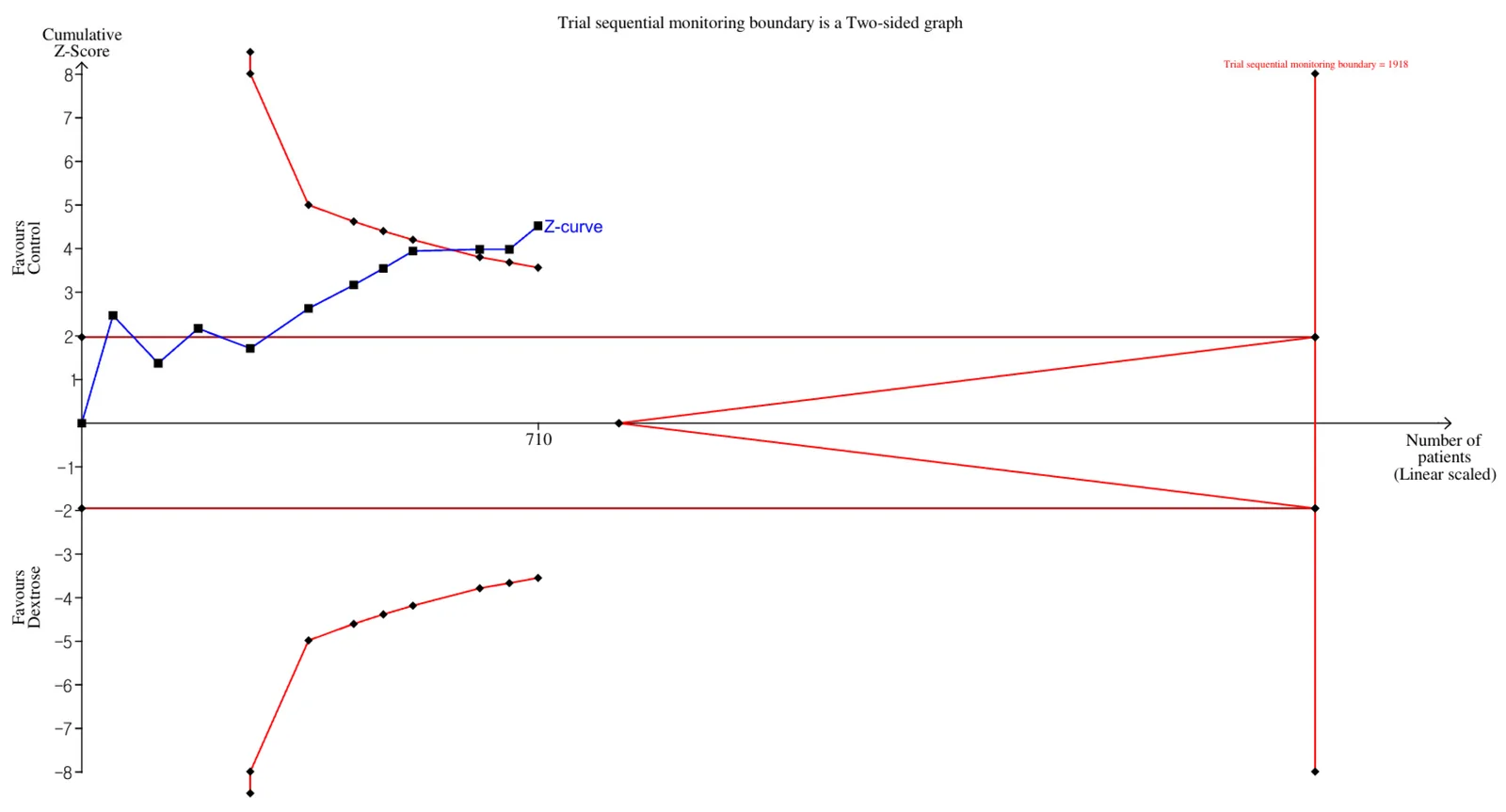
Trial sequential analysis (TSA) plot for the incidence of use of rescue antiemetics. The uppermost and lowermost continuous red lines represent the trial sequential monitoring boundaries for benefit and harm, respectively. The horizontal red lines indicate the conventional boundaries for statistical significance. The triangular red lines extending to the right represent the futility boundaries. The blue solid line represents the cumulative Z-curve, which crosses the trial sequential monitoring boundary, indicating that the finding remained statistically significant after adjustment for the risk of Type I error associated with repeated testing. This finding should be interpreted alongside the conventional meta-analysis, sensitivity analyses, and the corresponding GRADE certainty rating, rather than being regarded as definitive evidence that no further trials are needed. The vertical red line on the far right indicates the required information size (RIS) of 1918 patients.

TSA indicated that only 37.0% (710 of 1918 patients) of the RIS was accrued. The cumulative Z-curve crossed both the conventional test boundary and the trial sequential monitoring boundary (Figure 7).

### 3.7. Sensitivity and Subgroup Analyses

Because two outcomes with no specified time point contributed to the Overall PON, POV, and PONV syntheses from a single, unstratified or approximate assessment, a sensitivity analysis excluding these two studies was performed [34,35]. The direction and statistical significance of all three pooled estimates were unchanged (Overall PON: RR = 0.73, 95% CI 0.58–0.92, vs. full dataset RR = 0.73, 95% CI 0.62–0.87; Overall POV: RR = 1.03, 95% CI 0.53–2.01, vs. full dataset RR = 0.84, 95% CI 0.52–1.38, both non-significant; Overall PONV: RR = 0.59, 95% CI 0.43–0.82, vs. full dataset RR = 0.65, 95% CI 0.51–0.86) (Supplementary Table S2).

A Hartung–Knapp adjustment to the random-effects confidence intervals was applied as a sensitivity analysis for all ten outcomes. The confidence interval widened modestly for postoperative nausea, PONV, and rescue antiemetic outcomes, but narrowed for postoperative-vomiting outcomes, a recognized property of the Hartung–Knapp method when between-study heterogeneity is close to zero; the direction of statistical significance did not change for any outcome (Supplementary Table S2).

As a further sensitivity analysis, τ^2^ was re-estimated using restricted maximum likelihood (REML), combined with the Hartung–Knapp adjustment, and compared with the Paule–Mandel-based results. Results were nearly identical for outcomes with negligible heterogeneity (τ^2^ = 0 under both estimators for overall/early PON, POV, PONV, and late PON/POV). For the use of rescue antiemetics, the one outcome with meaningful heterogeneity (I^2^ = 36% under Paule–Mandel), REML estimated τ^2^ = 0, a known small-sample property of the REML estimator, which has been documented to underestimate between-study variance—sometimes truncating it to zero—when the number of studies is small; this is why Paule–Mandel is often recommended as the more robust choice in this setting. Despite this difference in τ^2^ estimation, the direction and statistical significance of the rescue-antiemetic estimate were unchanged (REML + HK: RR = 0.59, 95% CI 0.41–0.84, vs. Paule–Mandel + HK: RR = 0.57, 95% CI 0.35–0.92). The conclusions of this review are therefore robust to the choice between REML and Paule–Mandel for τ^2^ estimation (Supplementary Table S2).

To examine the potential influence of correlated comparisons derived from the same trial, a sensitivity analysis was performed retaining only one comparison per trial for the two trials contributing two sub-studies each from a shared control group (Özgüner 2025 [15], Salman 2020 [41]; the preoperative-only comparison was retained and the combined preoperative-and-intraoperative comparison was excluded for each). The direction and statistical significance of all ten pooled estimates were unchanged (Supplementary Table S2).

Across the ten outcomes analyzed, 6 of 98 sub-study comparisons had zero events in both arms and received the continuity correction described in Section 2.6.1; the remaining 22 comparisons had zero events in only one arm and were incorporated without correction (Supplementary Table S2).

Formal tests for subgroup interaction by administration timing (preoperative, preoperative + intraoperative, intraoperative, and postoperative) were computed for every outcome. These were statistically significant only for the use of rescue antiemetics (Q = 8.11, df = 3, *p* = 0.044); interaction tests for every PON, POV, and PONV outcome were not significant (all *p* > 0.4), indicating that the apparent timing-related differences in these outcomes are not formally supported and should be considered exploratory (Supplementary Table S2). A subgroup analysis by surgery type was interpretable only for overall PONV, given the small number of non-laparoscopic trials: laparoscopic surgery (k = 8) yielded RR = 0.55 (95% CI 0.39–0.79) and non-laparoscopic surgery (k = 2) yielded RR = 0.77 (95% CI 0.54–1.09), broadly similar in direction. For overall PON and POV, only one non-laparoscopic trial contributed data [35], precluding a meaningful comparison. A random-effects meta-regression of log(RR) against mean surgical duration was not significant for overall PON (*p* = 0.32), POV (*p* = 0.20), or PONV (*p* = 0.51), providing no evidence that surgical duration modifies the effect of perioperative dextrose in this dataset. A sensitivity analysis by specific anesthetic agent could not be performed, as all 13 included trials used general anesthesia without consistently reporting the specific induction or maintenance agents (Supplementary Table S2).

### 3.8. Publication Bias

Five outcomes (Overall PON, PONV, POV, Early PON, and PONV) included ≥ 10 sub-studies and were eligible for publication bias assessment. Across the funnel plot inspection, Egger’s test, trim-and-fill, selection models, PET-PEESE, and Bayesian sensitivity analyses, the pooled estimates remained consistent in direction and magnitude with the primary analyses for all outcomes (Supplementary Figures S22–S26, Supplementary Table S3).

### 3.9. Methodological Quality of Included Studies

Table 2 summarizes the risk-of-bias assessment. Overall, the included studies had either a low risk of bias [14,16,32,33,34,36,38], some concerns [15,31,35,39,40,41], or a high risk of bias [37]. Among the studies with some concerns, this was raised regarding only bias arising from the randomization process [15,31,35,39,40,41]. Rao (2017) [37], available to us only as a conference-abstract-form correspondence, was judged to be at high risk of bias overall because RoB2 signalling questions could not be answered across multiple domains due to insufficient methodological detail. The risk-of-bias judgments in Table 2 and the corresponding signalling-question detail in Supplementary Table S4 represent integrated study-level assessments rather than outcome-specific evaluations. We note this as a limitation: subjective, patient-reported outcomes such as nausea may be more susceptible to bias from imperfect blinding than more objective outcomes such as vomiting, and a single study-level judgment may not fully capture this distinction. Outcome-level RoB 2 assessments were not performed separately; instead, we applied an integrated study-level assessment, which may not fully capture potential differences in the risk of bias across individual outcomes.

### 3.10. Quality of Evidence

A concise Summary of Findings table for the primary outcomes is presented in Table 3; the complete GRADE evidence profile, including early- and late-phase and timing-subgroup results with full RoB2 signalling-question detail, is provided in Supplementary Tables S1 and S5.

The certainty of evidence was rated as moderate for overall PON, overall PONV, early PON, early PONV, and late PONV, reflecting a downgrade for risk of bias, as more than one-third of the pooled weight for each of these outcomes was contributed by trials judged ‘Some concerns’ for bias arising from the randomization process. The certainty of evidence was rated as low for the use of rescue antiemetics, reflecting downgrades for both risk of bias and inconsistency (moderate statistical heterogeneity and a significant subgroup interaction by administration timing). The certainty of evidence was rated as very low for overall POV, early POV, late PON, and late POV, reflecting downgrades for both risk of bias and imprecision (95% confidence intervals crossing the null value and spanning both a clinically important benefit and a clinically important harm (Table 3, Supplementary Table S5)).

## 4. Discussion

This systematic review and meta-analysis, incorporating trial sequential analysis (TSA), demonstrated that perioperative intravenous dextrose administration significantly reduces the incidence of overall and early PON, as well as overall, early, and late PONV. Furthermore, it was associated with a reduced need for rescue antiemetics in patients undergoing elective surgery under general anesthesia. Regarding the timing of administration, postoperative dextrose infusion consistently demonstrated antiemetic benefits for overall and early PON, as well as for overall and early PONV. In contrast, no significant effect was observed on late PONV in any subgroup. For the use of rescue antiemetics, efficacy was noted with preoperative administration alone, combined preoperative-and-intraoperative administration, and postoperative administration alone, whereas intraoperative administration alone showed no significant benefit.

The trial sequential analyses provide one component of the overall evidence assessment for these findings. The cumulative Z-curve crossed the trial sequential monitoring boundary for overall and early PON, overall PONV, and the use of rescue antiemetics, indicating that these findings remained statistically significant even after adjusting for the risk of Type I error from repeated significance testing. However, this should be interpreted alongside the corresponding GRADE certainty ratings (Table 3) and sensitivity analyses (Section 3.7) rather than as definitive proof that no further trials are needed, particularly given the multiplicity of outcomes tested. In contrast, for early and late PONV, the cumulative Z-curve did not reach the monitoring boundary, indicating that these specific outcomes require further investigation to confirm their statistical significance. No evidence of a difference was observed between the dextrose and control groups in the prevention of late PON or overall and late POV.

Overall, the findings of this study suggest that perioperative IV dextrose administration may serve as a useful nonpharmacological adjunct for the prevention of PONV. Several physiological mechanisms could explain this beneficial effect.

First, preoperative fasting induces a metabolic state characterized by reduced glucose availability and increased fat breakdown, leading to the production of ketone bodies. These metabolic changes may increase susceptibility to nausea and vomiting by enhancing the responsiveness of the central emetic pathways to perioperative stimuli such as opioids and volatile anesthetics [7]. Administration of intravenous dextrose may help restore metabolic balance and suppress ketone production, thereby reducing the activation of the central pathways involved in nausea and vomiting. In patients who are able to take oral fluids, shortened preoperative fasting and oral carbohydrate loading represent guideline-concordant options [42]. Intravenous dextrose administration may be considered a complementary or alternative strategy when preoperative oral intake is not feasible or is contraindicated, rather than a replacement for these established approaches.

Second, glucose administration stimulates endogenous insulin release and shifts the metabolism from a catabolic state to a more anabolic state. This metabolic effect may attenuate perioperative insulin resistance and reduce the physiological stress response to surgery [9,43]. Because surgical stress is associated with the release of inflammatory mediators that can activate the vagal afferent pathways involved in emesis, reducing this response may indirectly contribute to a lower incidence of PONV.

Third, maintaining stable blood glucose concentrations may help prevent autonomic nervous system disturbances associated with rapid glycemic fluctuations. Acute changes in blood glucose levels can increase sympathetic nervous system activity, which has been implicated in the development of nausea. The findings of Özgüner et al. [14] are noteworthy [15]. They observed that split-dose glucose administration, which resulted in more gradual changes in blood glucose levels than a single bolus dose, was associated with a lower incidence of PONV. This finding suggests that glycemic stability, rather than glucose concentration alone, may play an important role in the prevention of nausea and vomiting.

Taken together, perioperative dextrose administration may reduce PONV through multiple complementary mechanisms, including the restoration of metabolic homeostasis, attenuation of the surgical stress response, reduction in inflammatory activation, and maintenance of autonomic stability. These mechanisms may be particularly important during the early postoperative period, when fasting-induced metabolic disturbances are most prominent and potentially reversible.

However, the antiemetic effects of perioperative dextrose administration may vary according to patient characteristics. Although the overall findings of this meta-analysis were generally consistent with previous studies, sensitivity analyses showed that the significance for early postoperative nausea (PON) was lost after exclusion of the study by Demirdağ et al. [14], suggesting that the observed effects may have been influenced by specific patient populations.

Notably, the study by Demirdağ et al. included only patients with morbid obesity undergoing laparoscopic sleeve gastrectomy [14]. Although dextrose administration reduced early PONV, it was associated with a higher incidence of nausea and vomiting at 24 h and greater postoperative use of opioids and rescue antiemetics. The authors suggested that severe insulin resistance might impair glucose utilization and limit the metabolic benefits of dextrose administration. This explanation is biologically plausible because the proposed antiemetic effects of dextrose partly depend on the restoration of metabolic homeostasis. In patients with marked insulin resistance, glucose administration may contribute to perioperative hyperglycemia, which is associated with delayed gastric emptying and increased risk of PONV.

This discrepancy may be explained by the unique metabolic characteristics of the study population investigated by Demirdağ et al., which consisted exclusively of patients with morbid obesity [14]. In that study, the administration of dextrose-containing fluids after laparoscopic sleeve gastrectomy was associated with a transient reduction in early PONV. However, paradoxically, the incidence of nausea and vomiting at 24 h, as well as postoperative opioid and antiemetic requirements, was higher in the dextrose group. The authors proposed that severe insulin resistance in morbidly obese patients might impair normal glucose utilization, thereby contributing to the worsening of late postoperative symptoms. Taken together, these findings suggest that the efficacy of perioperative dextrose administration depends on the patient’s metabolic status. Therefore, caution is warranted when extrapolating these results to patients with morbid obesity or severe insulin resistance.

Interestingly, our findings suggest that the antiemetic efficacy of dextrose is significantly influenced by the timing of administration, with postoperative administration emerging as the most consistently effective strategy for reducing the incidence of PON and PONV. We propose that the clinical benefits of dextrose can be optimized when tailored to the unique metabolic and physiological requirements of each phase of the perioperative period. Intraoperative administration alone appears to have limited efficacy, likely because glucose is rapidly depleted as an immediate fuel source to meet the increased energy demands caused by surgical stress and anesthesia, leaving insufficient metabolic reserves to counteract the emetic triggers during the recovery period. In contrast, postoperative administration directly coincides with the period of peak fasting-induced metabolic disturbance and emetogenic stimulation, allowing dextrose to act as an immediate “resuscitation” measure that stabilizes the chemoreceptor trigger zone precisely when the risk of nausea and vomiting is highest—an effect that likely explains why postoperative dosing consistently reduced both overall and early PON and PONV in our analysis. Preoperative dextrose, by contrast, may act through a complementary “preload” mechanism that enhances physiological resistance to emetogenic stimuli before surgical stress begins. Notably, a reduced need for rescue antiemetics was observed across several regimens that included preoperative or postoperative dosing, namely preoperative administration alone, combined pre- and intraoperative administration, and postoperative administration alone, suggesting that multiple timing strategies, rather than intraoperative dosing in isolation, can meaningfully attenuate breakthrough emetic symptoms.

It should also be emphasized that the subgroup analyses by administration timing were not based on randomized comparisons and are potentially confounded by surgery type, patient population, dextrose dose and volume, fluid composition, baseline PONV risk, antiemetic prophylaxis, and anesthetic management; these timing-based findings should therefore be regarded as exploratory rather than confirmatory. Formal tests for subgroup interaction were significant only for the use of rescue antiemetics; interaction tests for PON, POV, and PONV were not statistically significant (all *p* > 0.4), indicating that the apparent differences in efficacy by timing for these outcomes are not formally supported and may reflect chance, confounding, or limited statistical power rather than a true difference in effect. In addition, because multiple outcomes, time windows, and subgroups were examined, the possibility of Type I error from multiple testing should be considered when interpreting individually significant findings.

With this caveat in mind, we offer the following as a hypothesis-generating, rather than confirmatory, interpretation of the observed patterns. Postoperative administration was associated with a reduced incidence of PON and PONV in our analysis. One possible explanation is that intraoperative administration alone may be metabolized rapidly during surgery, leaving insufficient metabolic reserves to counteract emetic triggers during recovery, whereas postoperative administration coincides more directly with the period of peak fasting-induced metabolic disturbance. Preoperative dextrose, by contrast, may act through a complementary mechanism that enhances physiological resistance to emetogenic stimuli before surgical stress begins. These mechanistic explanations were not directly tested in this meta-analysis and should be evaluated in future prospective studies rather than treated as established findings. Notably, a reduced need for rescue antiemetics was observed across several regimens that included preoperative or postoperative dosing—preoperative administration alone, combined pre- and intraoperative administration, and postoperative administration alone—consistent with the significant subgroup interaction test for this outcome; this timing-related pattern was not observed for PON, POV, or PONV.

Several methodological limitations of this study should be considered when interpreting the results. In practice, the reported time points varied considerably across studies (e.g., some studies reported outcomes at 0–3 h/3–6 h/6–24 h, others at PACU/~2 h/2–24 h, and others as a single combined assessment). When a study’s own reported stratification did not permit a clean assignment to either the early or late window—for example, McCaul et al., who reported outcomes only at PACU, ~2 h, and a combined 2–24 h period spanning both sides of the 6-h boundary—that study was not included in the corresponding window’s synthesis, rather than forcing a stratified split the original authors did not report. This reflects a limitation applied post hoc during data extraction rather than a pre-specified rule, and a supplementary table mapping every original study time point to the early, late, and overall analyses has been added for transparency (Supplementary Table S6). In addition, selecting the earliest reported time point within the early phase, while intended to minimize cumulative event counts, could introduce differential misclassification between groups; this is an inherent limitation of synthesizing heterogeneously time-stratified outcome data. Although all included studies used 5% dextrose solutions, substantial heterogeneity existed regarding administered volume, infusion rate, total dose, and timing of administration, which limited direct comparability across studies. In addition, baseline PONV risk, including Apfel risk scores, has not been consistently reported, making it difficult to determine whether differences in treatment effects reflect true physiological variations or simply differences in underlying patient risk. Furthermore, the included populations were clinically heterogeneous, ranging from relatively healthy women who underwent short-duration gynecologic laparoscopy to morbidly obese patients who underwent bariatric surgery. Consequently, the pooled estimates should be interpreted with caution when applied to specific patient subgroups. None of the included trials pre-specified exclusion of patients with diabetes, and perioperative blood glucose, insulin, sodium, or potassium concentrations were not consistently reported; therefore, the safety of dextrose administration in patients with diabetes or glucose intolerance could not be assessed in this review, and clinicians should monitor blood glucose when using perioperative dextrose in such patients until dedicated safety data become available.

Nevertheless, the low cost and physiological rationale of perioperative dextrose administration support its potential role as a component of multimodal PONV prevention strategies. Future studies should focus on identifying the optimal timing, dosing strategy, and target patient populations while carefully considering patient-specific metabolic factors such as obesity, insulin resistance, and perioperative glucose tolerance.

## 5. Conclusions

Perioperative intravenous dextrose may be a useful nonpharmacological adjunct for PONV prevention, with efficacy that appears to vary according to the timing of administration; this pattern was statistically supported only for the use of rescue antiemetics, not for PON, POV, or PONV. In this analysis, postoperative administration reduced the incidence of PON and PONV, and preoperative, combined preoperative-and-intraoperative, and postoperative administration each reduced the need for rescue antiemetics, whereas intraoperative administration alone showed no significant benefit. Because timing was not randomized and is confounded by surgery type, dose, and co-interventions, these timing-based findings should be regarded as exploratory, and the optimal timing, dose, and target population for perioperative dextrose warrant confirmation in future trials.

## Figures and Tables

**Table 1:** Characteristics of included studies.

Study (Author, Year)	Age (yrs)	Sex (M/F)	Height (cm)	Weight (kg)	ASA-PS (I/II)	Anesthesia	Duration (min)	Type of Surgery	Participants (D/C)		Dextrose Protocol	Duration of Infusion	Timing of Administration	Primary Outcome(s)
Cook (1990) [31]	32.2 ± 6.4	0/73	NR	61.7 ± 10.4	NR	GA	20.2 ± 6.1	Laparoscopy	25/24	Control (CSL)	LRS (20 mL/kg)	45 min	Intraoperative	Incidence of PONV (4-point scale)
										Intervention (CSL/dext)	5% Dextrose in LRS (20 mL/kg)			
McCaul (2003) [32]	32.8 ± 5.0	0/107	NR	63.3 ± 11.5	NR	GA	22.7 ± 6.7	GYN Laparoscopy	35/36	Control (CSL)	CSL (0.5 g/kg)	~20 min	After induction (intraoperative)	Incidence of PONV (VDS)
										Intervention (CSL/dext)	5% Dextrose in CSL (0.5 g/kg)			
Patel (2013) [33]	45.9 [43.4–48.4]	0/162	NR	27.0 [24.7–29.6] *	I–II	GA	153.5 [140.5–190.0]	GYN/Plastic/Breast	87/75	Control (LRS)	LRS (250 mL)	2 h	At surgical closure (intraoperative)	PONV severity (VRS, 0–10)
										Intervention (LRS/dext)	5% Dextrose in LRS (250 mL)			
Dabu-Bondoc (2013) [34]	37.7 ± 10.1	0/62	160.9 ± 7.7	69.1 ± 13.7	I–II	GA	100.0 ± 15.0	GYN Laparoscopy	30/32	Control (LRS)	LRS (1 L)	30 min	Upon PACU arrival (postoperative)	Nausea VRS score; rescue antiemetic use
										Intervention (LRS/dext)	5% Dextrose in LRS (1 L)			
Irkal (2016) [35]	29.1 ± 9.5	44/46	160.9 ± 9.8	59.0 ± 6.7	69/21	GA	119.2 ± 7.5	Middle-ear surgery	45/45	Control (LRS)	LRS (500 mL)	1 h	Upon PACU arrival (postoperative)	Incidence of PONV (~24 h)
										Intervention (LRS/dext)	5% Dextrose in LRS (500 mL)			
Mishra (2017) [36]	38.5 ± 11.8	28/72	159.2 ± 9.3	59.9 ± 9.9	I–II	GA	66.2 ± 16.0	Lap. Chole	50/50	Control (N/S)	N/S (250 mL)	2.5 h	When gallbladder was removed (intraoperative)	Incidence of PONV (binary)
										Intervention (LRS/dext)	5% Dextrose in LRS (250 mL)			
Rao (2017) [37]	43.5 ± 12.0	21/94	NR	24.5 ± 2.9 *	98/17	GA	72.5 ± 9.6	Lap. Chole	59/59	Control (LRS)	LRS (1000 mL)	30–40 min	Upon PACU arrival (postoperative)	PONV (VDS score)
										Intervention (LRS/dext)	5% Dextrose in LRS (1000 mL)			
Firouzian (2018) [38] ^a^	41.2 ± 12.7	0/121	NR	NR	NR	GA	75(65–85)	Lap. Chole	61/60	Control (LRS)	LRS (500 mL)	60 min	30 min before induction	PONV (VRS score)
										Intervention (LRS/dext)	5% Dextrose in LRS (500 mL)			
Atashkhoei (2018) [39]	31.3 ± 1.0	0/70	NR	68.4 ± 1.7	70/0	GA	58.0 ± 2.8	GYN Laparoscopy	35/35	Control (LRS)	LRS (10 mL/kg/h)	Variable	5 min before induction until end of surgery (preoperative–intraoperative)	Incidence of PONV (4-point scale)
										Intervention (LRS/dext)	5% Dextrose in LRS (10 mL/kg/h)			
Pin-on (2018) [40]	37.9 ± 9.6	0/66	NR	52.7 ± 9.1	70/16	GA	172.9 ± 60.4	GYN Laparoscopy	42/44	Control (NSS)	NSS (2 mL/kg/h)	Throughout operation	Intraoperative	Incidence of PONV
										Intervention (NSS/dext)	5% Dextrose in NSS (2 mL/kg/h)			
Salman (2020) [41]	48.8 ± 13.6	0/93	159.2 ± 6.5	75.8 ± 13.6	I–II	GA	57.0 ± 20.7	Lap. Chole	31/31 (preop); 31/31 (intraop)	Control (LRS)	LRS 10 mL/kg/h	30 min (preop)/Throughout op (intraop)	Group P: 30 min before induction (preoperative); Group I: Intraoperative	Incidence of PONV (VDS score)
										Intervention (LRS/dext) ‘preop’	Preop: 5% dextrose in 0.45% saline 10 mL/kg/h, Intraop: LRS 10 mL/kg/h			
										Intervention (LRS/dext) ‘intraop’	Preop: LRS 10 mL/kg/h Intraop: 5% dextrose in 0.45% saline 10 mL/kg/h,			
Liu (2024) [16]	45.4 ± 10.1	0/105	NR	24.3 ± 3.5 *	4/99	GA	130.8 ± 45.0	GYN Laparoscopy	55/50	Control (LRS)	LRS (400 mL)	30 min	Upon PACU arrival (postoperative)	Nausea (VRS score)
										Intervention (LRS/dext)	5% Dextrose in LRS (400 mL)			
Özgüner (2025) [15]	48.5 ± 9.9	43/47	NR	27.5 ± 2.9 *	I–II	GA	55.6 ± 8.1	Lap. Chole	30/30 (D); 30/30 (DD)	Control (NSS)	0.9% saline 400 mL (preop), 0.9% saline 10 mL/kg/h (intraop)	30 min each	Group D: 2 h before surgery (preoperative); Group DD: Split preoperative + intraoperative	24-h incidence of PONV (VDS score)
										Intervention (dext/NSS)	5% dextrose 400 mL (preop), 0.9% saline 10 mL/kg/h (intraop)			
										Intervention (dext/dext)	5% dextrose 200 mL (preop), 5% dextrose 200 mL (intraop) + 0.9% saline			
Demirdağ (2025) [14]	34.2 ± 10.4	66/135	167.0 ± 8.2	123.8 ± 24.9	III	GA	85.4 ± 15.1	Lap. Sleeve Gastrectomy	100/101	Control (NSS)	0.9% NSS (goal-directed, ~866 mL total)	Until end of surgery	Intraoperative	Incidence of PONV
										Intervention (NSS/dext)	0.9% NSS + 5% Dextrose (goal-directed, ~866 mL total)			

Values are presented as mean ± SD, mean [95% CI or range], or number of patients. (*) Weight data for these studies are reported as Body Mass Index (BMI, kg/m^2^). D/C, Dextrose group/Control group. ^a^ Assessed for eligibility and risk of bias but excluded from the quantitative meta-analysis because nausea and vomiting were reported only as continuous VRS (0–10) scores rather than as binary incidence data, precluding pooling with the risk-ratio framework used for the other 12 included trials. Abbreviations: ASA-PS, American Society of Anesthesiologists Physical Status; CSL, Compound Sodium Lactate; GA, General Anesthesia; GYN, Gynecologic; Lap. Chole, Laparoscopic Cholecystectomy; LRS, Lactated Ringer’s Solution; NR, Not Reported; NSS, Normal Saline Solution; PACU, Post-Anesthesia Care Unit; PONV, Postoperative Nausea and Vomiting; VDS, Verbal Descriptive Scale; VRS, Verbal Rating Scale.

**Table 2:** Risk of bias.

Author, Year	Bias Arising from the Randomization Process	Bias Due to Deviations from Intended Intervention	Bias Due to Missing Outcome Data	Bias in Measurement of the Outcome	Bias in Selection of the Reported Results	Overall Bias
Cook (1990) [31]	Some concern ^a^	Low risk ^b^	Low risk	Low risk	Low risk	Some concern
McCaul (2003) [32]	Low risk	Low risk	Low risk	Low risk	Low risk	Low risk
Patel (2013) [33]	Low risk	Low risk	Low risk	Low risk	Low risk	Low risk
Dabu-Bondoc (2013) [34]	Low risk	Low risk	Low risk	Low risk	Low risk	Low risk
Irkal (2016) [35]	Some concern ^a^	Low risk ^b^	Low risk	Low risk	Low risk	Some concern
Mishra (2017) [36]	Low risk	Low risk	Low risk	Low risk	Low risk	Low risk
Rao (2017) [37]	Some concern ^c^	Some concern ^c^	Some concern ^c^	Some concern ^c^	Some concern ^c^	High risk
Atashkhoei (2018) [39]	Some concern ^a^	Low risk ^b^	Low risk	Low risk	Low risk	Some concern
Firouzian (2018) [38]	Low risk	Low risk	Low risk	Low risk	Low risk	Low risk
Pin-on (2018) [40]	Some concern ^a^	Low risk ^b^	Low risk	Low risk	Low risk	Some concern
Salman (2020) [41]	Some concern ^a^	Low risk ^b^	Low risk	Low risk	Low risk	Some concern
Liu (2024) [16]	Low risk	Low risk	Low risk	Low risk	Low risk	Low risk
Özgüner (2025) [15]	Some concern ^a^	Low risk ^b^	Low risk	Low risk	Low risk	Some concern
Demirdağ (2025) [14]	Low risk	Low risk	Low risk	Low risk	Low risk	Low risk

^a^ We classified the bias arising from the randomization process domain as “some concern” because there was no information regarding allocation concealment. ^b^ We classified the bias due to deviations from the intended intervention as “low risk” because even if the personnel knew, it was not expected to affect the intended intervention, and analysis was not performed in the wrong group in the study. ^c^ Because Rao (2017) [37] was reported only as a conference-abstract-form correspondence, RoB2 signalling questions across all domains could not be answered due to insufficient methodological detail; per RoB2 guidance, unanswerable signalling questions default to a judgment of ‘Some concerns’ rather than being left unclassified.

**Table 3:** Summary of Findings Table.

Outcome	Anticipated Absolute Risk (Control)	Anticipated Absolute Risk (Dextrose)	Relative Effect (95% CI)	№ of Participants (Studies)/Certainty	Comments
Postoperative nausea (PON), overall	409 per 1000	299 per 1000 (254 to 356). Risk difference: 110 fewer per 1000 (53 to 155 fewer)	RR 0.73 (0.62–0.87)	856 (11 sub-studies) ⊕⊕⊕◯ Moderate	Dextrose likely reduces overall PON
Postoperative vomiting (POV), overall	75 per 1000	64 per 1000 (38 to 106). Risk difference: 11 fewer per 1000 (37 fewer to 32 more)	RR 0.84 (0.52–1.38)	856 (11 sub-studies) ⊕⊕◯◯ Low ^a^	The evidence is very uncertain about the effect of dextrose on overall POV
Postoperative nausea and vomiting (PONV), overall	297 per 1000	193 per 1000 (151 to 247). Risk difference: 104 fewer per 1000 (50 to 146 fewer)	RR 0.65 (0.51–0.86)	743 (10 sub-studies) ⊕⊕⊕◯ Moderate	Dextrose likely reduces overall PONV
Use of rescue antiemetics	315 per 1000	180 per 1000 (117 to 274). Risk difference: 135 fewer per 1000 (41 to 198 fewer)	RR 0.57 (0.37–0.87)	710 (11 sub-studies) ⊕⊕⊕◯ Moderate	Dextrose likely reduces rescue antiemetic use

GRADE Working Group grades of evidence. ⊕⊕⊕⊕ = High certainty; ⊕⊕⊕◯ = Moderate certainty; ⊕⊕◯◯ = Low certainty; ⊕◯◯◯ = Very low certainty. High certainty: we are very confident that the true effect lies close to that of the estimate of the effect. Moderate certainty: the true effect is likely to be close to the estimate of the effect, but there is a possibility that it is substantially different. Low certainty: the true effect may be substantially different from the estimate of the effect. Very low certainty: the true effect is likely to be substantially different from the estimate of the effect. ^a^ Downgraded one level for imprecision: the 95% confidence interval crosses the null value and includes both a clinically important benefit and a clinically important harm; trial sequential analysis indicates only 9.7% of the required information size has been accrued.

## Data Availability

The data presented in this study is available on request from the corresponding author. Any requests will be reviewed against compliance with ethical, scientific, regulatory, and legal requirements. Requests to access the datasets should be directed to roman00@naver.com.

## Supplementary Materials

The following supporting information can be downloaded at https://www.mdpi.com/article/10.3390/medicina62091718/s1, Search Strategy; Supplementary Table S1: Trial Sequential Analysis Parameters by Outcome; Supplementary Table S2: Sensitivity Analyses; Supplementary Table S3: Sensitivity analyses for publication bias across outcomes with ≥10 sub-studies; Supplementary Table S4: RoB2 Signalling-Question Judgments by Study; Supplementary Table S5: The GRADE evidence quality for each outcome; Supplementary Table S6: PRISMA checklist; Supplementary Table S7: Mapping of Original Study Time Points to Early/Late/Overall Analyses Supplementary; Figures S1–S7: Sensitivity (leave-one-out) analyses and trial sequential analysis plots for overall and early/late postoperative nausea (PON); Figures S8–S13: Forest plots, sensitivity analyses, and trial sequential analysis plots for overall and late postoperative vomiting (POV); Figures S14–S20: Sensitivity analyses, forest plots, and trial sequential analysis plots for overall, early, and late postoperative nausea and vomiting (PONV); Figure S21: Sensitivity analysis for use of rescue antiemetics; Figures S22–S26: Sensitivity analyses comparing frequentist and Bayesian publication-bias adjustment methods for overall PON, overall PONV, overall POV, early PON, and early PONV.

## Abbreviations

The following abbreviations are used in this manuscript:

PONV	Postoperative Nausea and Vomiting
PON	Postoperative Nausea
POV	Postoperative Vomiting
RCT	Randomized Controlled Trial
TSA	Trial Sequential Analysis
RR	Risk Ratio
CI	Confidence Interval
