# Supplementary material for: The Effect of Intravenous Dextrose Administration on Postoperative Nausea and Vomiting: Systematic Review and Meta-Analysis with Trial Sequential Analysis
Source: Medicina (Kaunas). 2026 Sep 7;62(9):1718. doi: 10.3390/medicina62091718 (PMC13609844; doi:10.3390/medicina62091718)

Search Strategy

Databases (PubMed, Ovid-EMBASE, Cochrane CENTRAL) and Google Scholar were searched from database inception through the end of February 2026. The ClinicalTrials.gov registry and the OpenSIGLE grey-literature database were searched over the same period.

PubMed

#1 "Postoperative Nausea and Vomiting"[Mesh]

#2 "postoperative nausea and vomiting"[tiab] OR PONV[tiab]

#3 "Nausea"[Mesh] OR nausea[tiab]

#4 "Vomiting"[Mesh] OR vomiting[tiab]

#5 #1 OR #2 OR #3 OR #4

#6 "Glucose"[Mesh] OR dextrose[tiab] OR glucose[tiab]

#7 "carbohydrate solution"[tiab] OR "carbohydrate loading"[tiab]

#8 #6 OR #7

#9 "Randomized Controlled Trial"[pt] OR randomized[tiab] OR randomised[tiab] OR "RCT"[tiab] OR randomly[tiab]

#10 #5 AND #8 AND #9

Ovid-EMBASE

1. exp postoperative nausea and vomiting/
2. (postoperative nausea and vomiting or PONV).tw,kw.
3. exp nausea/ or nausea.tw,kw.
4. exp vomiting/ or vomiting.tw,kw.
5. 1 or 2 or 3 or 4
6. exp glucose/ or (dextrose or glucose).tw,kw.

7. (carbohydrate solution or carbohydrate loading).tw,kw.

8. 6 or 7

9. exp randomized controlled trial/ or (randomized or randomised or RCT or randomly).tw,kw.

10. 5 and 8 and 9

Cochrane Central Register of Controlled Trials (CENTRAL)

#1 MeSH descriptor: [Postoperative Nausea and Vomiting] explode all trees

#2 ("postoperative nausea and vomiting" OR PONV):ti,ab,kw

#3 MeSH descriptor: [Nausea] explode all trees OR nausea:ti,ab,kw

#4 MeSH descriptor: [Vomiting] explode all trees OR vomiting:ti,ab,kw

#5 #1 OR #2 OR #3 OR #4

#6 MeSH descriptor: [Glucose] explode all trees OR (dextrose OR glucose):ti,ab,kw

#7 ("carbohydrate solution" OR "carbohydrate loading"):ti,ab,kw

#8 #6 OR #7

#9 #5 AND #8

Google Scholar

("postoperative nausea and vomiting" OR PONV) AND (dextrose OR "carbohydrate solution") AND (randomized OR randomised OR RCT)

The first 200 results, ranked by relevance, were screened; Google Scholar does not support field tags or MeSH/Emtree indexing.

OpenSIGLE (grey literature)

("postoperative nausea and vomiting" OR PONV) AND (dextrose OR "carbohydrate solution")

(Same free-text terms as the Google Scholar search, applied to OpenSIGLE's basic search interface, which does not support field tags or Boolean nesting beyond AND/OR.)

ClinicalTrials.gov (trial registry)

Condition/disease: "postoperative nausea and vomiting" OR PONV

Other terms: dextrose OR "carbohydrate solution"

Study type: Interventional studies (Randomized)

Supplementary Table S1: Trial Sequential Analysis Parameters by Outcome

TSA software: Copenhagen Trial Unit TSA, version 0.9.5.10. Studies ordered chronologically by publication year. Alpha=5% (two-sided), Power=80%, assumed relative risk reduction=20%.

Outcome	k	Control event %	D^2	RIS (fixed)	RIS (adjusted)	%RIS accrued
Overall PON	11	40.9%	0%	1084	1084	79.0%
Overall POV	11	7.5%	0%	8799	8799	9.7%
Overall PONV	10	29.7%	0%	1737	1737	42.8%
Early PON	10	38.2%	0%	1205	1205	63.5%
Early POV	11	7.5%	0%	8799	8799	9.7%
Early PONV	10	27.6%	0%	1918	1918	38.7%
Late PON	8	18.1%	0%	3269	3269	19.4%
Late POV	8	3.7%	0%	18549	18549	3.4%
Late PONV	8	17.3%	0%	3434	3434	16.7%
Rescue antiemetics	11	31.5%	36%	1596	2492	28.5%

k = number of sub-studies contributing; D^2 = diversity; RIS = required information size.

Supplementary Table S2: Sensitivity Analyses

Hartung-Knapp adjustment

Outcome	RR	Classic 95% CI	Hartung-Knapp 95% CI
Overall PON	0.73	0.62–0.87	0.59–0.90
Overall POV	0.84	0.52–1.38	0.58–1.22
Overall PONV	0.65	0.51–0.86	0.49–0.86
Early PON	0.73	0.60–0.88	0.59–0.90
Early POV	0.84	0.52–1.38	0.58–1.22
Early PONV	0.66	0.51–0.85	0.50–0.87
Late PON	0.83	0.54–1.28	0.49–1.41
Late POV	1.09	0.48–2.47	0.70–1.670
Late PONV	0.56	0.36–0.85	0.33–0.95
Rescue antiemetics	0.57	0.37–0.87	0.35–0.93

REML + Hartung–Knapp adjustment (compared with Paule–Mandel + Hartung–Knapp)

Outcome	Paule–Mandel + HK RR [95% CI]	REML + HK RR [95% CI]	τ^2 (PM / REML)
Overall PON	0.73 [0.61–0.87]	0.73 [0.61–0.87]	0 / 0
Overall POV	0.85 [0.58–1.26]	0.85 [0.58–1.26]	0 / 0
Overall PONV	0.65 [0.50–0.86]	0.65 [0.50–0.86]	0 / 0
Early PON	0.73 [0.59–0.90]	0.73 [0.59–0.90]	0 / 0
Early POV	0.85 [0.58–1.26]	0.85 [0.58–1.26]	0 / 0
Early PONV	0.66 [0.50–0.87]	0.66 [0.50–0.87]	0 / 0
Late PON	0.95 [0.63–1.44]	0.84 [0.49–1.42]	0 / 0
Late POV	1.09 [0.70–1.69]	1.09 [0.70–1.69]	0 / 0
Late PONV	0.58 [0.39–0.86]	0.58 [0.39–0.86]	0 / 0
Rescue antiemetics	0.57 [0.35–0.92]	0.59 [0.41–0.84]	0.2556 / 0

For rescue antiemetic use — the only outcome with meaningful heterogeneity under Paule–Mandel (I^2=36%) — REML estimated τ^2=0, a known small-sample property of the REML estimator (which can underestimate, and sometimes truncate to zero, the between-study variance when the number of studies is small). Despite this difference in τ^2, the direction and statistical significance of every outcome, including rescue antiemetic use, were unchanged between the two methods.

Sensitivity analysis: one comparison per trial (multi-arm correlation)

Salman (2020) and Özgüner (2025) each contributed two correlated sub-study comparisons drawn from a shared control group (preoperative-only, and combined preoperative-and-intraoperative). To assess whether this

correlation materially affects the pooled estimates, a sensitivity analysis was performed retaining only the preoperative-only comparison for each of these two trials and excluding the combined preoperative-and-intraoperative comparison, across all ten outcomes.

**Outcome**	**Full dataset RR [95% CI]**	**One comparison per trial RR [95% CI]**	**Conclusion**
Overall PON	0.73 [0.62–0.87]	0.72 [0.61–0.86]	Unchanged
Overall POV	0.85 [0.51–1.42]	0.86 [0.51–1.45]	Unchanged
Overall PONV	0.65 [0.51–0.83]	0.64 [0.49–0.82]	Unchanged
Early PON	0.73 [0.60–0.88]	0.72 [0.59–0.87]	Unchanged
Early POV	0.85 [0.51–1.42]	0.86 [0.51–1.45]	Unchanged
Early PONV	0.66 [0.51–0.85]	0.64 [0.50–0.83]	Unchanged
Late PON	0.95 [0.67–1.35]	1.01 [0.70–1.45]	Unchanged
Late POV	1.09 [0.48–2.47]	1.10 [0.48–2.53]	Unchanged
Late PONV	0.58 [0.37–0.90]	0.62 [0.39–0.99]	Unchanged
Rescue antiemetics	0.57 [0.37–0.87]	0.65 [0.44–0.94]	Unchanged

The direction and statistical significance of every pooled estimate were unchanged, indicating that the results are not materially driven by the correlated-comparisons structure of these two trials.

Zero-event cell handling across all outcomes

**Category**	**N (of 98 sub-study comparisons)**	**Handling**
Double-zero (both arms 0 events)	6	Continuity correction of 0.1 added to each cell prior to pooling
Single-zero (one arm 0 events)	22	Incorporated into the exact Mantel-Haenszel pooled estimate without correction
No zero cells	70	N/A

Double-zero comparisons: Mishra (2017) [Overall PONV, Early PONV], Cook (1990), Özgüner (2025, combined preop+intraop comparison), Salman (2020, both comparisons) [all in Late POV].

Exclusion of Dabu-Bondoc (2013) and Irkal (2016) from Overall outcomes

**Outcome**	**Full dataset**	**Excluding Dabu/Irkal**	**Conclusion**
Overall PON	RR=0.73 [0.62–0.87]	RR=0.73 [0.58–0.92]	Unchanged (significant)
Overall POV	RR=0.84 [0.52–1.38]	RR=1.03 [0.53–2.01]	Unchanged (non-significant)
Overall PONV	RR=0.65 [0.51–0.86]	RR=0.59 [0.43–0.82]	Unchanged (significant)

Subgroup interaction tests (by administration timing)

**Outcome**	**Q (df=3)**	**p-value**	**Interpretation**	

Overall PON	0.70	0.874	Not significant	
Overall POV	0.80	0.850	Not significant	
Overall PONV	0.88	0.831	Not significant	
Early PON	1.01	0.798	Not significant	
Early POV	0.80	0.850	Not significant	
Early PONV	0.80	0.850	Not significant	
Late PON	2.77	0.429	Not significant	
Late POV	0.44	0.933	Not significant	
Late PONV	1.06	0.787	Not significant	
Rescue antiemetics	8.11	0.044	Significant	

Surgery type and surgical duration

Surgery-type subgroup (laparoscopic vs. non-laparoscopic) was only interpretable for overall PONV: laparoscopic k=8, RR=0.55 [0.39–0.79]; non-laparoscopic k=2, RR=0.77 [0.54–1.09]. For overall PON and POV only one non-laparoscopic trial (Irkal 2016) contributed data, precluding comparison.

Random-effects meta-regression of log(RR) on mean surgical duration: overall PON β=−0.0032 (p=0.32); overall POV β=−0.0090 (p=0.20); overall PONV β=0.0027 (p=0.51). No significant association with duration was found for any outcome.

Supplementary Table S3. Sensitivity analyses for publication bias across outcom[illegible]

Outcome	Conventional RE	Trim-Fill	PET	PEESE	Sel. Model S1	Sel. Model S2	Sel. Model S3	Bayes Baseline	Bayes S1 (2×)	Bayes S2 (3×)	([illegible]
**Overall PON**	0.73 (0.62–0.87)	0.72 (0.61–0.85)	0.64 (0.51–0.82)	0.72 (0.61–0.85)	0.73 (0.62–0.87)	0.73 (0.62–0.87)	0.73 (0.62–0.86)	0.73 (0.62–0.87)	0.70 (0.61–0.81)	0.69 (0.61–0.77)	0[illegible]
**Overall PONV**	0.65 (0.51–0.83)	0.65 (0.51–0.83)	0.64 (0.43–0.96)	0.65 (0.50–0.84)	0.65 (0.51–0.83)	0.65 (0.51–0.83)	0.65 (0.51–0.83)	0.65 (0.51–0.83)	0.62 (0.50–0.76)	0.59 (0.49–0.72)	0[illegible]
**Overall POV**	0.84 (0.52–1.38)	0.84 (0.52–1.38)	0.90 (0.43–1.87)	0.88 (0.57–1.38)	0.84 (0.52–1.38)	0.84 (0.52–1.38)	0.83 (0.51–1.34)	0.85 (0.53–1.37)	0.85 (0.53–1.37)	0.85 (0.53–1.37)	0[illegible]
**Early PON**	0.73 (0.60–0.88)	0.72 (0.57–0.91)	0.64 (0.49–0.83)	0.71 (0.58–0.86)	0.73 (0.60–0.88)	0.73 (0.60–0.88)	0.73 (0.60–0.88)	0.73 (0.60–0.88)	0.70 (0.60–0.81)	0.68 (0.60–0.77)	0[illegible]
**Early PONV**	0.66 (0.51–0.85)	0.66 (0.51–0.85)	0.66 (0.44–1.00)	0.66 (0.50–0.86)	0.66 (0.50–0.86)	0.66 (0.50–0.86)	0.66 (0.51–0.85)	0.66 (0.51–0.85)	0.62 (0.50–0.77)	0.60 (0.49–0.72)	0[illegible]

Values are pooled risk ratios (RR) with 95% confidence (or credible) intervals. Frequentist analyses include the conventional random-effects [illegible] regression-based adjustments, and three Vevea–Hedges selection models (S1–S3, reflecting increasingly strong assumptions about selective publica[illegible] Bayesian random-effects model, three publication-bias-adjusted models with progressively stronger downweighting of small/non-significant stud[illegible] alternative prior specifications (Very Weak, Skeptical, Very Skeptical). Across all five outcomes, pooled estimates were consistent in direction an[illegible] RE) analyses. PON, postoperative nausea; PONV, postoperative nausea and vomiting; POV, postoperative vomiting; RE, random-effects; PET, p[illegible] estimate with standard error.

Supplementary Table S4: RoB2 Signalling-Question Judgments by Study

Domain 1: Bias arising from the randomization process

**Study**	**1.1 Random sequence?**	**1.2 Allocation concealed?**	**1.3 Baseline imbalance?**	**D1 judgment**
Cook (1990)	Yes	No information	No	Some concerns
McCaul (2003)	Yes	Yes	No	Low risk
Dabu-Bondoc (2013)	Yes	Yes	No	Low risk
Patel (2013)	Yes	Yes	No	Low risk
Irkal (2016)	Yes	No information	No	Some concerns
Mishra (2017)	Yes	Yes	No	Low risk
Atashkhoei (2018)	Yes	No information	No	Some concerns
Firouzian (2018)	Yes	Yes	No	Low risk
Pin-on (2018)	Yes	No information	No	Some concerns
Salman (2020)	Yes	No information	No	Some concerns
Liu (2024)	Yes	Yes	No	Low risk
Özgüner (2025)	Yes	No information	No	Some concerns
Demirdağ (2025)	Yes	Yes	No	Low risk

Domain 2: Bias due to deviations from intended interventions

Study	2.1 Participants aware?	2.2 Personnel aware?	2.3 Deviations occurred?	2.6 Correct-group analysis?	D2 judgment
Cook (1990)	No	Yes/Probably yes	No	Yes	Low risk
McCaul (2003)	No	No	N/A	Yes	Low risk
Dabu-Bondoc (2013)	No	No	N/A	Yes	Low risk
Patel (2013)	No	No	N/A	Yes	Low risk
Irkal (2016)	No	Yes/Probably yes	No	Yes	Low risk
Mishra (2017)	No	No	N/A	Yes	Low risk
Atashkhoei (2018)	No	Yes/Probably yes	No	Yes	Low risk
Firouzian (2018)	No	No	N/A	Yes	Low risk
Pin-on (2018)	No	Yes/Probably yes	No	Yes	Low risk
Salman (2020)	No	Yes/Probably yes	No	Yes	Low risk
Liu (2024)	No	No	N/A	Yes	Low risk
Özgüner (2025)	No	Yes/Probably yes	No	Yes	Low risk
Demirdağ (2025)	No	No	N/A	Yes	Low risk

Domains 3–5 and overall judgment

Study	D3 (missing	D4 (measurement)	D5 (selective	Overall

	data)		reporting)	
Cook (1990)	Low risk	Low risk	Low risk	Some concerns
McCaul (2003)	Low risk	Low risk	Low risk	Low risk
Dabu-Bondoc (2013)	Low risk	Low risk	Low risk	Low risk
Patel (2013)	Low risk	Low risk	Low risk	Low risk
Irkal (2016)	Low risk	Low risk	Low risk	Some concerns
Mishra (2017)	Low risk	Low risk	Low risk	Low risk
Atashkhoei (2018)	Low risk	Low risk	Low risk	Some concerns
Firouzian (2018)†	Low risk	Low risk	Low risk	Low risk
Pin-on (2018)	Low risk	Low risk	Low risk	Some concerns
Salman (2020)	Low risk	Low risk	Low risk	Some concerns
Liu (2024)	Low risk	Low risk	Low risk	Low risk
Özgüner (2025)	Low risk	Low risk	Low risk	Some concerns
Demirdağ (2025)	Low risk	Low risk	Low risk	Low risk

† Firouzian (2018): risk of bias assessed and reported here, but the trial was excluded from quantitative synthesis (continuous VRS outcome, not binary incidence).

Supplementary Table S5: The GRADE evidence quality for each outcome.

Outcome	Timepoint (measured)	Timepoint (administration)	No of studies	No of patients	Quality assessment					Quality
					ROB	Inconsistency	Indirectness	Imprecision	Publication bias	
PON	Overall	Combined	9	856	serious [a]	not serious	not serious	not serious	not serious	⊕⊕⊕◯ Moderate
		Pre	2	91	serious [a]	not serious	not serious	serious [b]	**NA**	⊕◯◯◯ Very low
		Pre+Intra	2	91	serious [a]	serious [a]	not serious	serious [b]	**NA**	⊕◯◯◯ Very low
		Intra	4	407	serious [a]	not serious	not serious	serious [b]	**NA**	⊕◯◯◯ Very low
		Post	3	267	serious [a]	not serious	not serious	serious [b]	**NA**	⊕◯◯◯ Very low
	Early	Combined	8	766	serious [a]	not serious	not serious	not serious	not serious	⊕⊕⊕◯ Moderate
		Pre	2	91	serious [a]	not serious	not serious	serious [b]	**NA**	⊕◯◯◯ Very low
		Pre+Intra	2	91	serious [a]	serious [a]	not serious	serious [b]	**NA**	⊕◯◯◯ Very low
		Intra	4	407	serious [a]	not serious	not serious	serious [b]	**NA**	⊕◯◯◯ Very low
		Post	2	177	serious [a]	not serious	not serious	serious [b]	**NA**	⊕◯◯◯ Very low
	Late	Combined	6	633	serious [a]	not serious	not serious	not serious	**NA**	⊕⊕◯◯ Low
		Pre	2	91	serious [a]	serious [a]	not serious	serious [b]	**NA**	⊕◯◯◯ Very low

		Pre+Intra	2	122	serious [a]	not serious	not serious	serious [b]	**NA**	⊕◯◯◯ Very low
		Intra	3	336	serious [a]	not serious	not serious	serious [b]	**NA**	⊕◯◯◯ Very low
		Post	1	115	serious [a]	**NA**	not serious	serious [b]	**NA**	⊕◯◯◯ Very low
POV	overall	Combined	9	856	serious [a]	not serious	not serious	**serious[c]**	not serious	⊕⊕◯◯ Low
		Pre	2	91	serious [a]	not serious	not serious	serious [b]	**NA**	⊕◯◯◯ Very low
		Pre+Intra	2	91	serious [a]	not serious	not serious	serious [b]	**NA**	⊕◯◯◯ Very low
		Intra	4	407	serious [a]	not serious	not serious	serious [b]	**NA**	⊕◯◯◯ Very low
		Post	3	205	serious [a]	not serious	not serious	serious [b]	**NA**	⊕◯◯◯ Very low
	late	Combined	6	633	serious [a]	not serious	not serious	not serious	**NA**	⊕⊕◯◯ Low
		Pre	2	91	serious [a]	not serious	not serious	serious [b]	**NA**	⊕◯◯◯ Very low
		Pre+Intra	2	91	serious [a]	not serious	not serious	serious [b]	**NA**	⊕◯◯◯ Very low
		Intra	3	336	serious [a]	not serious	not serious	serious [b]	**NA**	⊕◯◯◯ Very low
		Post	1	115	serious [a]	**NA**	not serious	serious [b]	**NA**	⊕◯◯◯ Very low
PONV	overall	Combined	8	743	serious [a]	not serious	not serious	not serious	not serious	⊕⊕⊕◯ Moderate
		Pre	3	161	serious [a]	not serious	not serious	serious [b]	**NA**	⊕◯◯◯ Very low

		Pre+Intra	2	91	serious [a]	serious [a]	not serious	serious [b]	**NA**	⊕◯◯◯ Very low
		Intra	2	181	serious [a]	not serious	not serious	serious [b]	**NA**	⊕◯◯◯ Very low
		Post	3	310	serious [a]	not serious	not serious	serious [b]	**NA**	⊕◯◯◯ Very low
	Early	Combined	8	743	serious [a]	not serious	not serious	not serious	not serious	⊕⊕⊕◯ Moderate
		Pre	3	161	serious [a]	not serious 0	not serious	serious [b]	**NA**	⊕◯◯◯ Very low
		Pre+Intra	2	91	serious [a]	serious [a]	not serious	serious [b]	**NA**	⊕◯◯◯ Very low
		Intra	2	181	serious [a]	not serious	not serious	serious [b]	**NA**	⊕◯◯◯ Very low
		Post	3	310	serious [a]	not serious	not serious	serious [b]	**NA**	⊕◯◯◯ Very low
	late	Combined	6	572	serious [a]	not serious	not serious	not serious	**NA**	⊕⊕◯◯ Low
		Pre	3	161	serious [a]	not serious	not serious	serious [b]	**NA**	⊕◯◯◯ Very low
		Pre+Intra	2	91	serious [a]	not serious	not serious	serious [b]	**NA**	⊕◯◯◯ Very low
		Intra	1	100	serious [a]	**NA**	not serious	serious [b]	**NA**	⊕◯◯◯ Very low
		Post	2	220	serious [a]	not serious	not serious	serious [b]	**NA**	⊕◯◯◯ Very low
**Use of RA**		Combined	9	710	serious [a]	not serious	not serious	not serious	not serious	⊕⊕⊕◯ Moderate
		Pre	3	161	serious [a]	not serious	not serious	serious [b]	**NA**	⊕◯◯◯ Very low

	Pre+Intra	2	91	serious [a]	not serious	not serious	serious [b]	**NA**	⊕◯◯◯ Very low
	Intra	3	211	serious [a]	not serious	not serious	serious [b]	**NA**	⊕◯◯◯ Very low
	Post	3	257	serious [a]	not serious	not serious	serious [b]	**NA**	⊕◯◯◯ Very low

GRADE Working Group grades of evidence
High certainty: we are very confident that the true effect lies close to that of the estimate of the effect.
Moderate certainty: we are moderately confident in the effect estimate: the true effect is likely to be close to the estimate of the effect, but there is a possibility that it is substantially different.
Low certainty: our confidence in the effect estimate is limited: the true effect may be substantially different from the estimate of the effect.
Very low certainty: we have very little confidence in the effect estimate: the true effect is likely to be substantially different from the estimate of effect.

[a] We downgraded risk of bias by one level for outcomes where more than one-third of the pooled weight was contributed by trials judged 'Some concerns' for Domain 1 (randomization process), reflecting uncertainty about allocation concealment across a substantial proportion of the evidence base.

[b] We downgraded the certainty of evidence by one level for inconsistency due to heterogeneity among studies, with an I^2 statistic over 50% or P_{chi2} less than 0.05.

[c] We downgraded imprecision by one level for serious imprecision due to very wide confidence intervals, including both substantial harms and benefits, or small numbers of studies and patients.

Supplementary Table S6: PRISMA checklist

Section and Topic	Item #	Checklist item	Location where item is reported
**TITLE**			
Title	1	Identify the report as a systematic review.	#1
**ABSTRACT**			
Abstract	2	See the PRISMA 2020 for Abstracts checklist.	#1-2
**INTRODUCTION**			
Rationale	3	Describe the rationale for the review in the context of existing knowledge.	#2-3
Objectives	4	Provide an explicit statement of the objective(s) or question(s) the review addresses.	#3
**METHODS**			
Eligibility criteria	5	Specify the inclusion and exclusion criteria for the review and how studies were grouped for the syntheses.	#4
Information sources	6	Specify all databases, registers, websites, organisations, reference lists and other sources searched or consulted to identify studies. Specify the date when each source was last searched or consulted.	#4-5
Search strategy	7	Present the full search strategies for all databases, registers and websites, including any filters and limits used.	#4-5
Selection process	8	Specify the methods used to decide whether a study met the inclusion criteria of the review, including how many reviewers screened each record and each report retrieved, whether they worked independently, and if applicable, details of automation tools used in the process.	#5
Data collection process	9	Specify the methods used to collect data from reports, including how many reviewers collected data from each report, whether they worked independently, any processes for obtaining or confirming data from study investigators, and if applicable, details of automation tools used in the process.	#5
Data items	10a	List and define all outcomes for which data were sought. Specify whether all results that were compatible with each outcome domain in each study were sought (e.g. for all measures, time points, analyses), and if not, the methods used to decide which results to collect.	#5
	10b	List and define all other variables for which data were sought (e.g. participant and intervention characteristics, funding sources). Describe any assumptions made about any missing or unclear information.	#5
Study risk of bias assessment	11	Specify the methods used to assess risk of bias in the included studies, including details of the tool(s) used, how many reviewers assessed each study and whether they worked independently, and if applicable, details of automation tools used in the process.	#7
Effect measures	12	Specify for each outcome the effect measure(s) (e.g. risk ratio, mean difference) used in the synthesis or presentation of results.	#6
Synthesis methods	13a	Describe the processes used to decide which studies were eligible for each synthesis (e.g. tabulating the study intervention characteristics and comparing against the planned groups for each synthesis (item #5)).	#6-7
	13b	Describe any methods required to prepare the data for presentation or synthesis, such as handling of missing summary statistics, or data conversions.	#6-7
	13c	Describe any methods used to tabulate or visually display results of individual studies and syntheses.	#6-7

Section and Topic	Item #	Checklist item	Location where item is reported
	13d	Describe any methods used to synthesize results and provide a rationale for the choice(s). If meta-analysis was performed, describe the model(s), method(s) to identify the presence and extent of statistical heterogeneity, and software package(s) used.	#6-7
	13e	Describe any methods used to explore possible causes of heterogeneity among study results (e.g. subgroup analysis, meta-regression).	#6-7
	13f	Describe any sensitivity analyses conducted to assess robustness of the synthesized results.	#6-7
Reporting bias assessment	14	Describe any methods used to assess risk of bias due to missing results in a synthesis (arising from reporting biases).	#6-7
Certainty assessment	15	Describe any methods used to assess certainty (or confidence) in the body of evidence for an outcome.	#7-8
**RESULTS**			
Study selection	16a	Describe the results of the search and selection process, from the number of records identified in the search to the number of studies included in the review, ideally using a flow diagram.	#9, Figure 1
	16b	Cite studies that might appear to meet the inclusion criteria, but which were excluded, and explain why they were excluded.	#9, Figure 1
Study characteristics	17	Cite each included study and present its characteristics.	#10-13, Table 1
Risk of bias in studies	18	Present assessments of risk of bias for each included study.	#22-24, Table 2
Results of individual studies	19	For all outcomes, present, for each study: (a) summary statistics for each group (where appropriate) and (b) an effect estimate and its precision (e.g. confidence/credible interval), ideally using structured tables or plots.	#13-22
Results of syntheses	20a	For each synthesis, briefly summarise the characteristics and risk of bias among contributing studies.	#13-22
	20b	Present results of all statistical syntheses conducted. If meta-analysis was done, present for each the summary estimate and its precision (e.g. confidence/credible interval) and measures of statistical heterogeneity. If comparing groups, describe the direction of the effect.	#13-22
	20c	Present results of all investigations of possible causes of heterogeneity among study results.	#13-22
	20d	Present results of all sensitivity analyses conducted to assess the robustness of the synthesized results.	#13-22
Reporting biases	21	Present assessments of risk of bias due to missing results (arising from reporting biases) for each synthesis assessed.	#22, Supplementary Table 3
Certainty of evidence	22	Present assessments of certainty (or confidence) in the body of evidence for each outcome assessed.	#24-25, Supplementary Table S
**DISCUSSION**			
Discussion	23a	Provide a general interpretation of the results in the context of other evidence.	#25
	23b	Discuss any limitations of the evidence included in the review.	#27-28

Section and Topic	Item #	Checklist item	Location where item is reported
	23c	Discuss any limitations of the review processes used.	#27-28
	23d	Discuss implications of the results for practice, policy, and future research.	#28
**OTHER INFORMATION**			
Registration and protocol	24a	Provide registration information for the review, including register name and registration number, or state that the review was not registered.	#4
	24b	Indicate where the review protocol can be accessed, or state that a protocol was not prepared.	#4
	24c	Describe and explain any amendments to information provided at registration or in the protocol.	#4
Support	25	Describe sources of financial or non-financial support for the review, and the role of the funders or sponsors in the review.	#29
Competing interests	26	Declare any competing interests of review authors.	#29
Availability of data, code and other materials	27	Report which of the following are publicly available and where they can be found: template data collection forms; data extracted from included studies; data used for all analyses; analytic code; any other materials used in the review.	#29

From: Page MJ, McKenzie JE, Bossuyt PM, Boutron I, Hoffmann TC, Mulrow CD, et al. The PRISMA 2020 statement: an updated guideline for reporting systematic reviews. BMJ 2021;372:n71. doi: 10.1136/bmj.n71. This work is licensed under CC BY 4.0. To view a copy of this license, visit https://creativecommons.org/licenses/by/4.0/

Supplementary Table S7: Mapping of Original Study Time Points to Early/Late/Overall Analyses

This table lists, for each included trial, the postoperative assessment time point(s) as originally reported, the outcome(s) reported at each time point, and how these were mapped to the early (0–6 h), late (6–48 h), or overall (unspecified time point) analyses in this review.

Study	Time point(s) as originally reported	Outcome(s) reported	Mapped to
Cook (1990)	Pre-op; 0–3 h; 3–24 h; 24–48 h; 48–72 h	Nausea, Vomiting (separate)	0–3 h → Early; 24–48 h → Late; 3–24 h not used (spans boundary, not assignable)
McCaul (2003)	PACU; ~2 h; 2–24 h	Nausea, Vomiting, Antiemetic use, Overall	PACU → Early; 2–24 h not used (spans 6-h boundary, not assignable); no Late/Overall contribution
Patel (2013)	On arrival; 1–30 min; 31–60 min; 61–120 min	PONV (combined)	On arrival → Early; no Late/Overall time point reported
Dabu-Bondoc (2013)	Single assessment (no time-stratified breakdown)	Nausea, Vomiting (separate)	→ Early and Overall (same single value used for both, per Methods rule for unspecified time points)
Irkal (2016)	Single assessment (~24 h, approximate)	PONV (combined), Vomiting	→ Early and Overall (same single value used for both)
Mishra (2017)	~30 min; 30–60 min; 60–90 min; 90–120 min; 2–6 h; 6–24 h; Overall	PONV (combined)	~30 min → Early; 6–24 h → Late; Overall (as reported) → Overall
Atashkhoei (2018)	PACU; 0–3 h; 3–6 h; 6–24 h; Overall	PONV (combined)	PACU → Early; 6–24 h → Late; Overall (as reported) → Overall
Firouzian (2018)†	PACU (immediate); 30/60/90/120 min; 6 h; 12 h; 24 h	Nausea VRS, Vomiting score (continuous)	Not applicable — excluded from quantitative synthesis (continuous outcome)
Pin-on (2018)	0, 30, 60, 90, 120 min; 4, 8, 12, 16, 20, 24 h	Nausea, Retching, Vomiting (separate)	0 min → Early; 24 h → Late
Rao (2017)	0–6 h; 6–24 h	Nausea, Vomiting, PONV (combined)	0–6 h → Early; 6–24 h → Late
Liu (2024)	0–1 h; 1–3 h; 3–6 h; 6–12 h; 12–24 h	PONV (combined)	0–1 h → Early; 6–12 h → Late
Salman (2020)*	Not confirmed against original publication	PON, POV, PONV, Rescue antiemetics	Early/Late/Overall assignment as used in the analysis dataset; original time-point wording to be confirmed
Özgüner (2025)*	Not confirmed against original publication	PON, POV, PONV, Rescue antiemetics	Early/Late/Overall assignment as used in the analysis dataset; original time-point wording to be confirmed
Demirdağ (2025)*	Not confirmed against original publication	PON, POV, PONV, Rescue antiemetics	Early/Late/Overall assignment as used in the analysis dataset; original time-

			point wording to be confirmed

† Firouzian (2018) was assessed for eligibility and risk of bias but excluded from the quantitative (binary RR) synthesis because it reported only continuous VRS nausea/vomiting scores.

** The original time-point wording for Salman (2020), Özgüner (2025), and DemirdÃ (2025) has not yet been re-confirmed against the original publications for this table; the Early/Late/Overall assignments used in the meta-analysis are correct and unaffected, but the exact reported time-point language should be added here before final submission.*

Supplementary Figure S1. Sensitivity analysis removing each study at a time for the incidence of overall postoperative nausea (PON). Each study is represented by a filled square proportional to its sample size, with horizontal lines indicating the 95% confidence interval (CI) of the mean difference. The black diamond denotes the overall pooled effect estimate with its 95% CI.

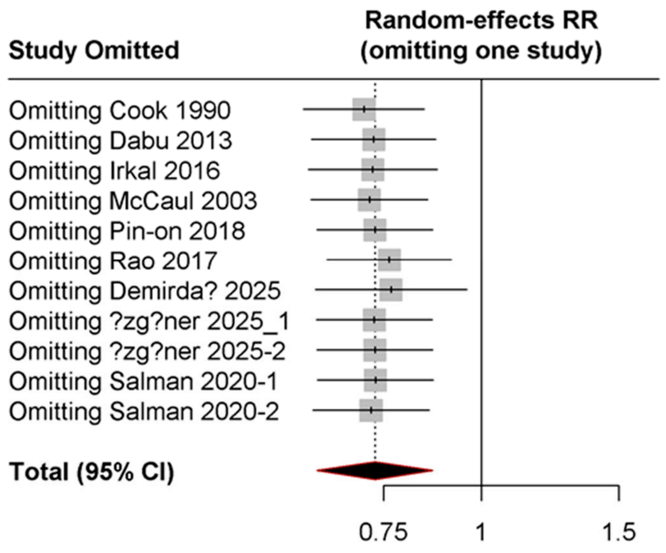

Supplementary Figure S2. Forest plot for the incidence of early postoperative nausea (PON) stratified by the timing of dextrose administration. Each study is represented by a filled square proportional to its weight, with horizontal lines indicating the 95% confidence interval (CI). The black diamonds represent the subgroup-specific pooled risk ratios (RRs), and the open diamond at the bottom indicates the overall pooled RR for all included studies. The red horizontal bar represents the prediction interval. Vertical dashed lines represent the null effect (RR = 1).

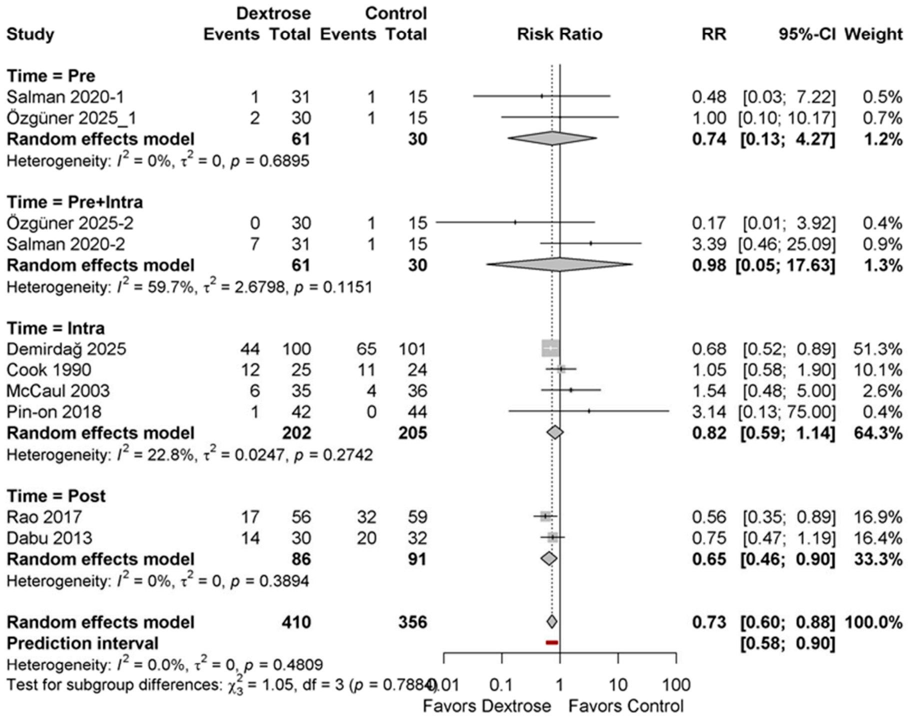

Supplementary Figure S3. Sensitivity analysis removing each study at a time for the incidence of early postoperative nausea (PON). Each study is represented by a filled square proportional to its sample size, with horizontal lines indicating the 95% confidence interval (CI) of the mean difference. The black diamond denotes the overall pooled effect estimate with its 95% CI.

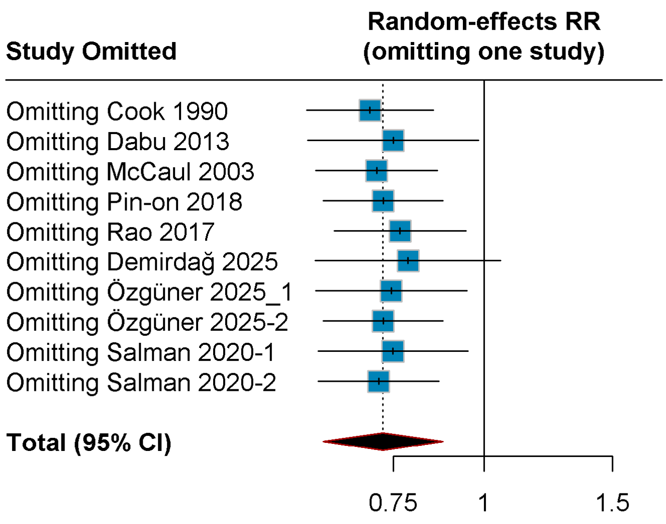

Supplementary Figure S4. Trial sequential analysis (TSA) plot for the incidence of early postoperative nausea (PON). The uppermost and lowermost continuous red lines represent the trial sequential monitoring boundaries for benefit and harm, respectively. The horizontal red lines indicate the conventional boundaries for statistical significance. The triangular red lines extending to the right represent the futility boundaries. The blue solid line with square markers represents the cumulative Z-curve, which crosses the conventional test boundary and the trial sequential monitoring boundary, indicating that the evidence is conclusive. The vertical red line on the far right indicates the required information size (RIS) of 1214 patients.

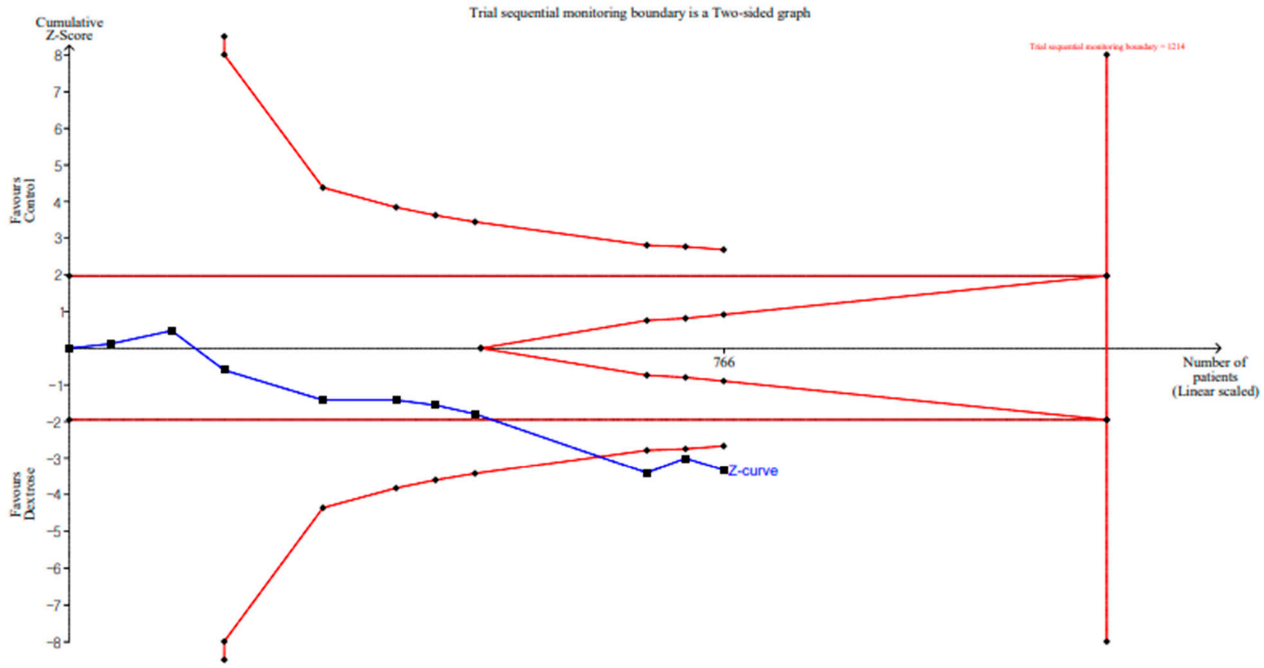

Supplementary Figure S5. Forest plot for the incidence of late postoperative nausea (PON) stratified by the timing of dextrose administration. Each study is represented by a filled square proportional to its weight, with horizontal lines indicating the 95% confidence interval (CI). The black diamonds represent the subgroup-specific pooled risk ratios (RRs), and the open diamond at the bottom indicates the overall pooled RR for all included studies. The red horizontal bar represents the prediction interval. Vertical dashed lines represent the null effect (RR = 1).

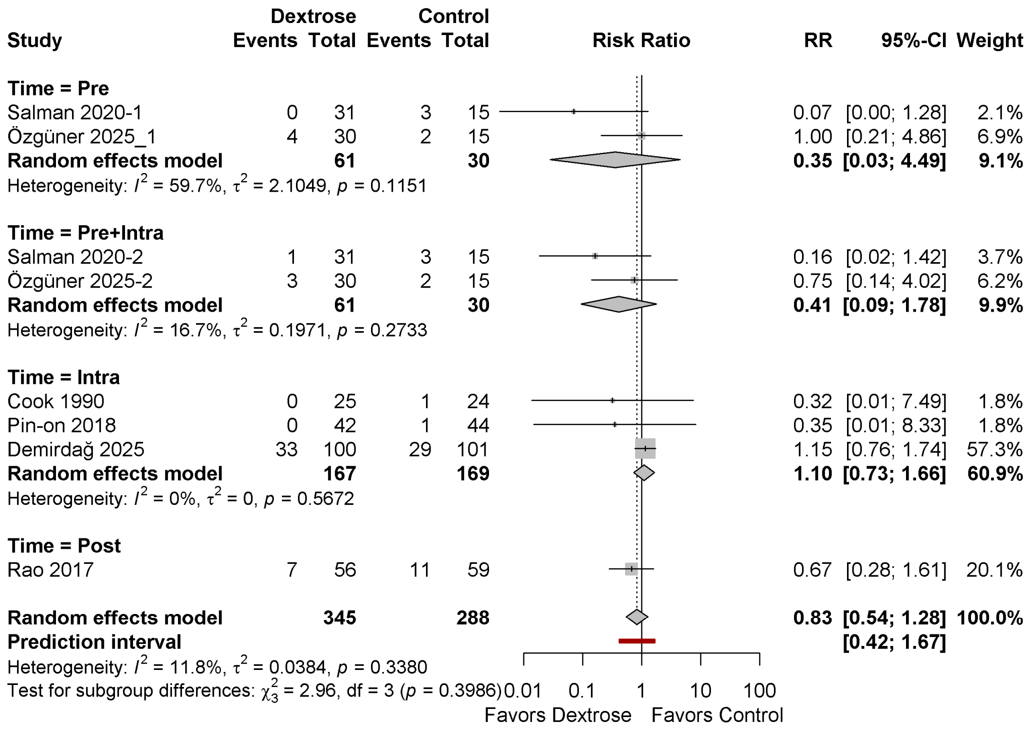

Supplementary Figure S6. Sensitivity analysis removing each study at a time for the incidence of late postoperative nausea (PON). Each study is represented by a filled square proportional to its sample size, with horizontal lines indicating the 95% confidence interval (CI) of the mean difference. The black diamond denotes the overall pooled effect estimate with its 95% CI.

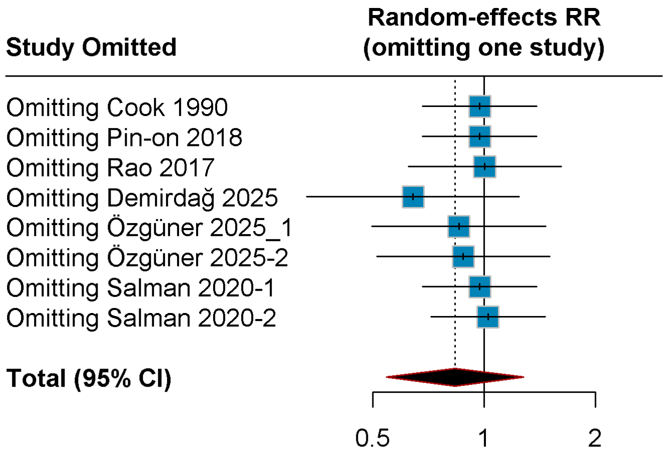

Supplementary Figure S7. Trial sequential analysis (TSA) plot for the incidence of late postoperative nausea (PON). The uppermost and lowermost continuous red lines represent the trial sequential monitoring boundaries for benefit and harm, respectively. The horizontal red lines indicate the conventional boundaries for statistical significance. The triangular red lines extending to the right represent the futility boundaries. The blue solid line represents the cumulative Z-curve, which did not cross any of the monitoring boundaries, indicating that the current evidence is inconclusive and requires further investigation. The vertical red line on the far right indicates the required information size (RIS) of 5784 patients.

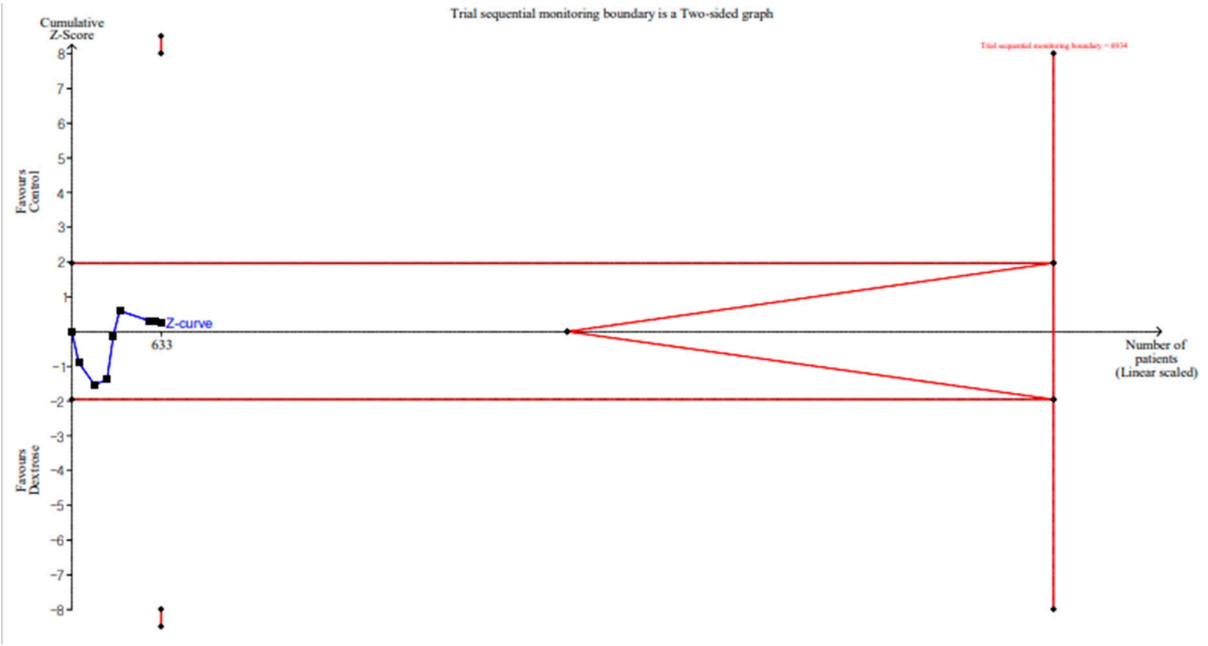

Supplementary Figure S8. Forest plot for the incidence of overall postoperative vomiting (POV). Each study is represented by a filled square proportional to its weight, with horizontal lines indicating the 95% confidence interval (CI). The open diamond at the bottom represents the overall pooled risk ratio (RR) for all included studies. Vertical dashed line represents the null effect (RR = 1).

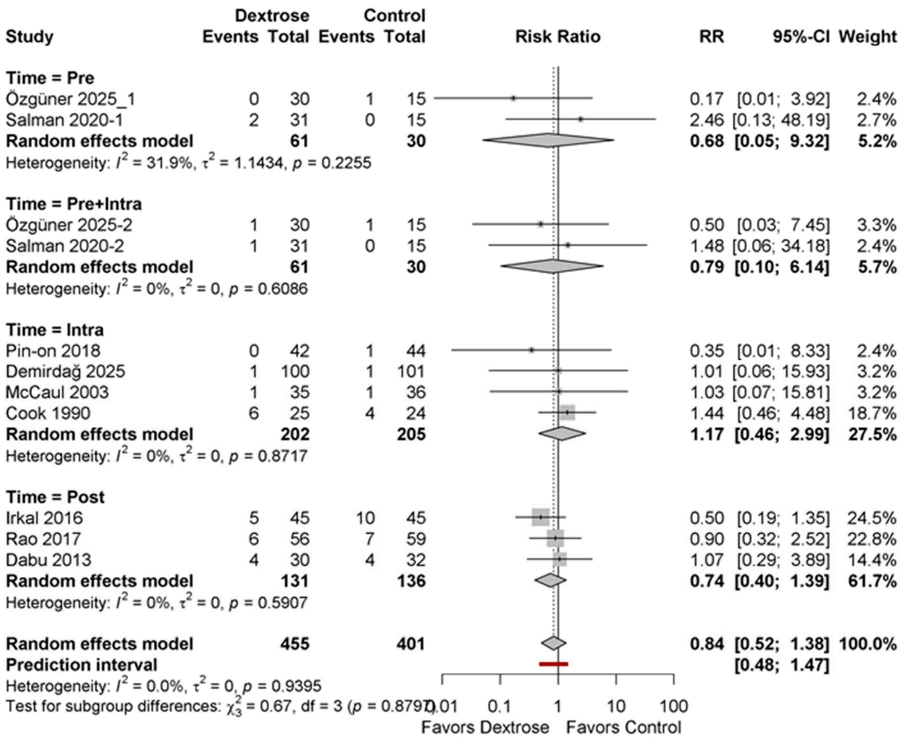

Supplementary Figure S9. Sensitivity analysis removing each study at a time for the incidence of overall postoperative vomiting (POV). Each study is represented by a filled square proportional to its sample size, with horizontal lines indicating the 95% confidence interval (CI) of the mean difference. The black diamond denotes the overall pooled effect estimate with its 95% CI.

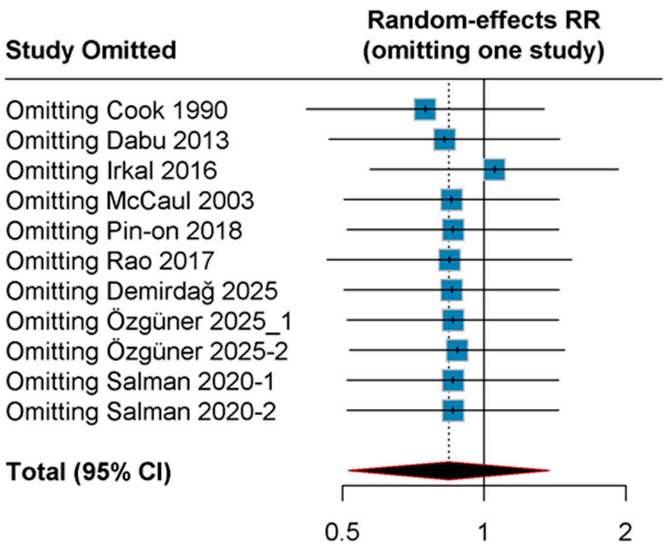

Supplementary Figure S10 Trial sequential analysis (TSA) plot for the incidence of overall postoperative vomiting (POV). The uppermost and lowermost continuous red lines represent the trial sequential monitoring boundaries for benefit and harm, respectively. The horizontal red lines indicate the conventional boundaries for statistical significance. The triangular red lines extending to the right represent the futility boundaries. The blue solid line represents the cumulative Z-curve, which remained within the monitoring boundaries and did not reach the RIS of 8783 patients (as indicated on the far right), suggesting that the current evidence is inconclusive and requires further investigation.

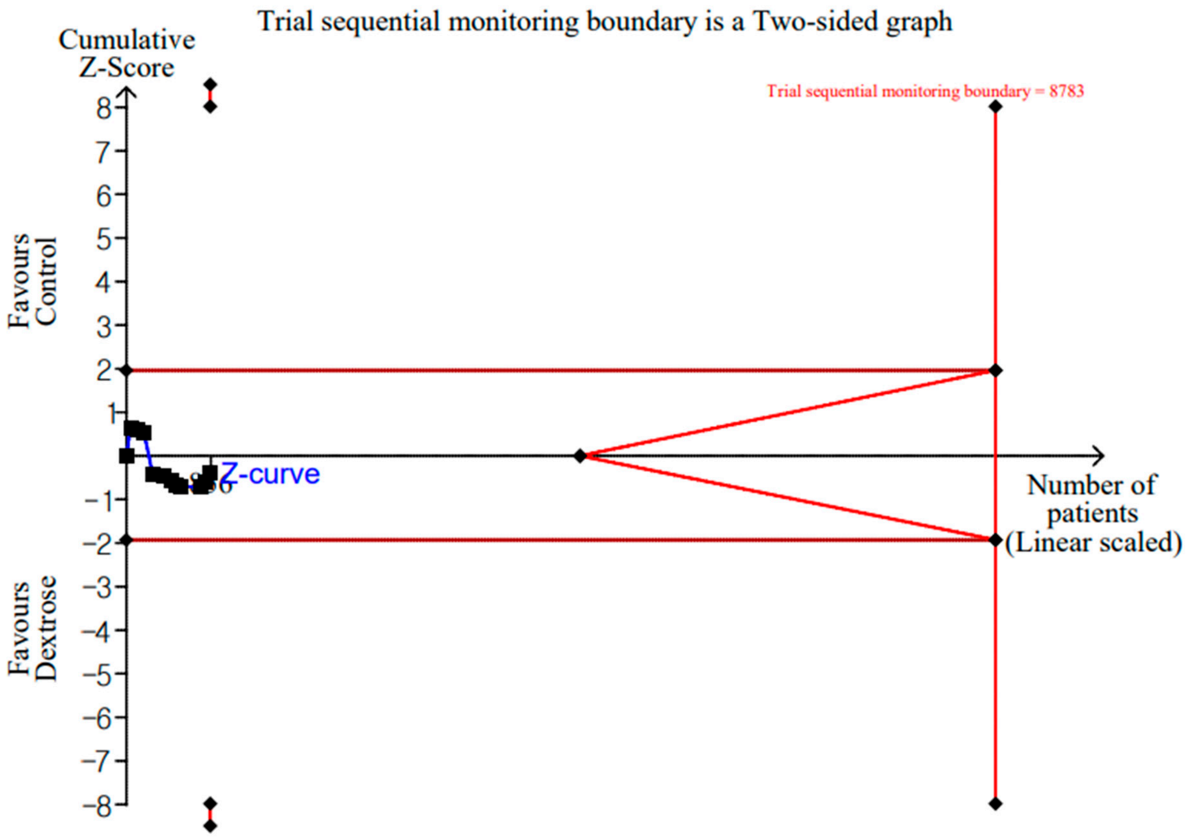

Supplementary Figure S11. Forest plot of the incidence of late postoperative vomiting (POV). Each study is represented by a filled square proportional to its weight, with horizontal lines indicating the 95% confidence interval (CI). The open diamond at the bottom represents the overall pooled risk ratio (RR) for all included studies. The vertical dashed line represents the null effect (RR = 1).

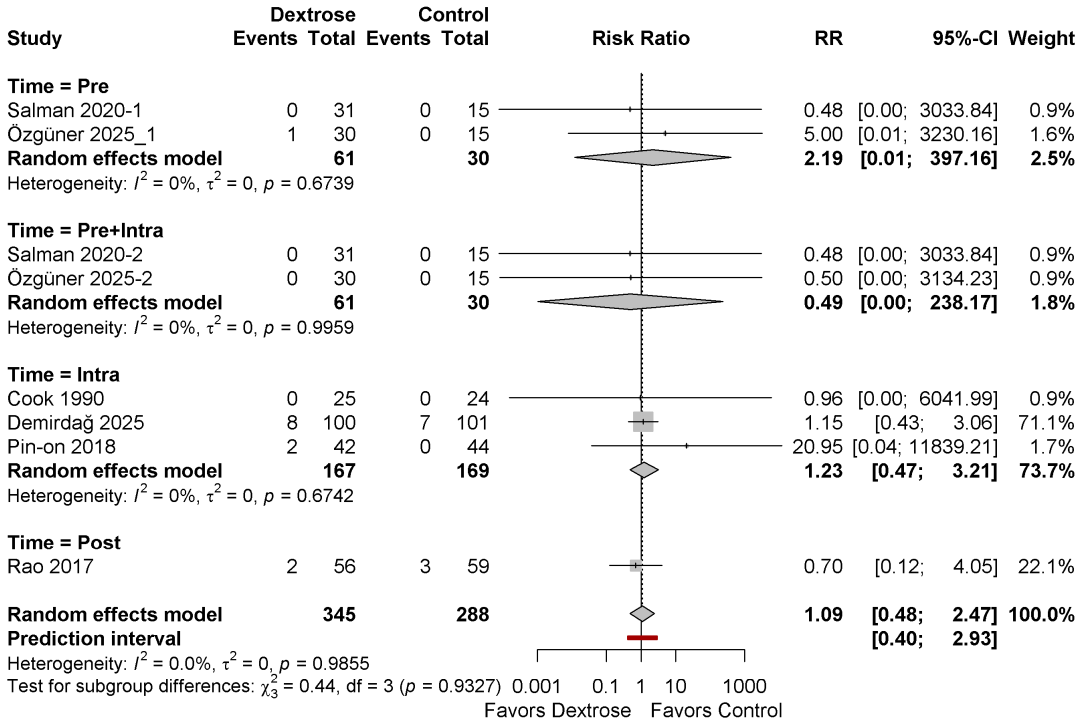

Supplementary Figure S12. Sensitivity analysis removing each study at a time for the incidence of late postoperative vomiting (POV). Each study is represented by a filled square proportional to its sample size, with horizontal lines indicating the 95% confidence interval (CI) of the mean difference. The black diamond denotes the overall pooled effect estimate with its 95% CI.

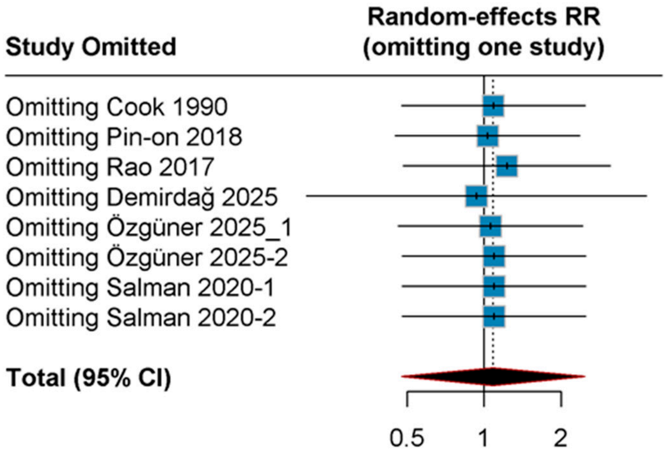

Supplementary Figure S13. Trial sequential analysis (TSA) plot for the incidence of late postoperative vomiting (POV). The horizontal red lines indicate the conventional boundaries for statistical significance. The cumulative Z-curve (blue solid line) remained within the monitoring boundaries, indicating that the current evidence is inconclusive and lacks sufficient power to demonstrate a clear treatment effect.

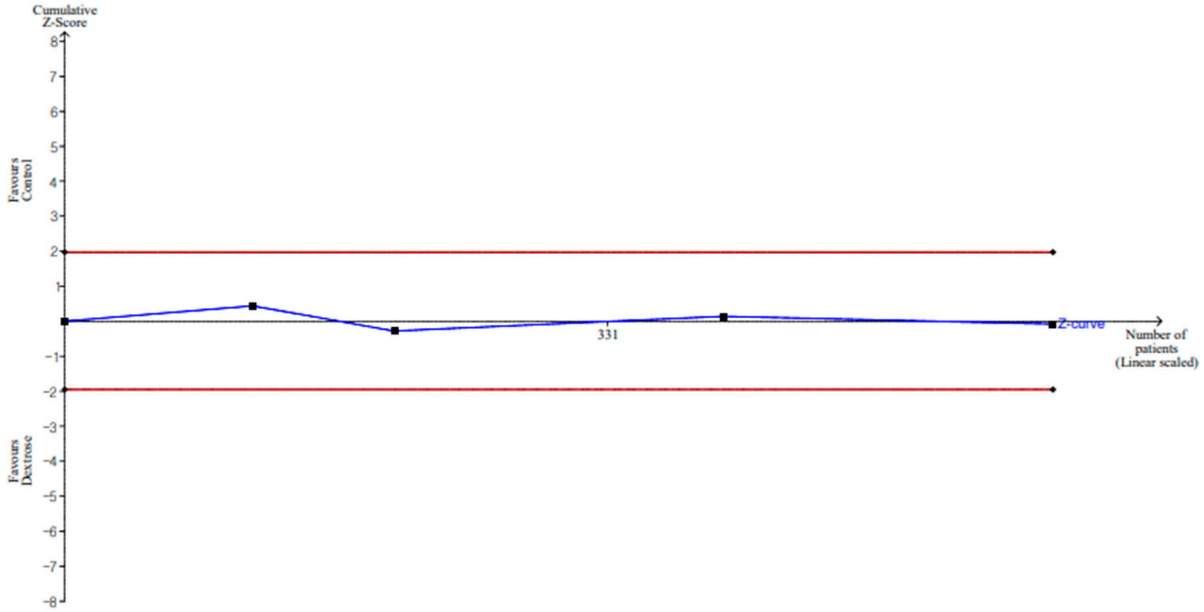

Supplementary Figure S14. Sensitivity analysis removing each study at a time for the incidence of overall postoperative nausea and vomiting (PONV). Each study is represented by a filled square proportional to its sample size, with horizontal lines indicating the 95% confidence interval (CI) of the mean difference. The black diamond denotes the overall pooled effect estimate with its 95% CI.

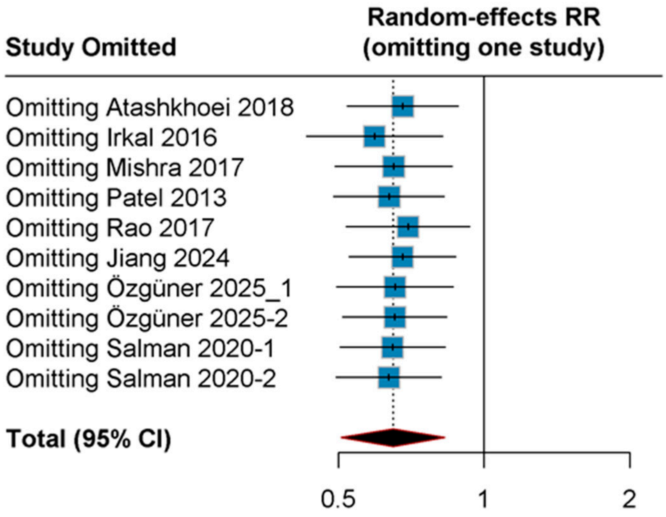

Supplementary Figure S15. Forest plot of the incidence of early postoperative nausea and vomiting (PONV) stratified by the timing of dextrose administration. Each study is represented by a filled square proportional to its weight, with horizontal lines indicating the 95% confidence interval (CI). The black diamonds represent the subgroup-specific pooled risk ratios (RRs), and the open diamond at the bottom indicates the overall pooled RR for all included studies. The red horizontal bar represents the prediction interval. Vertical dashed lines represent the null effect (RR = 1).

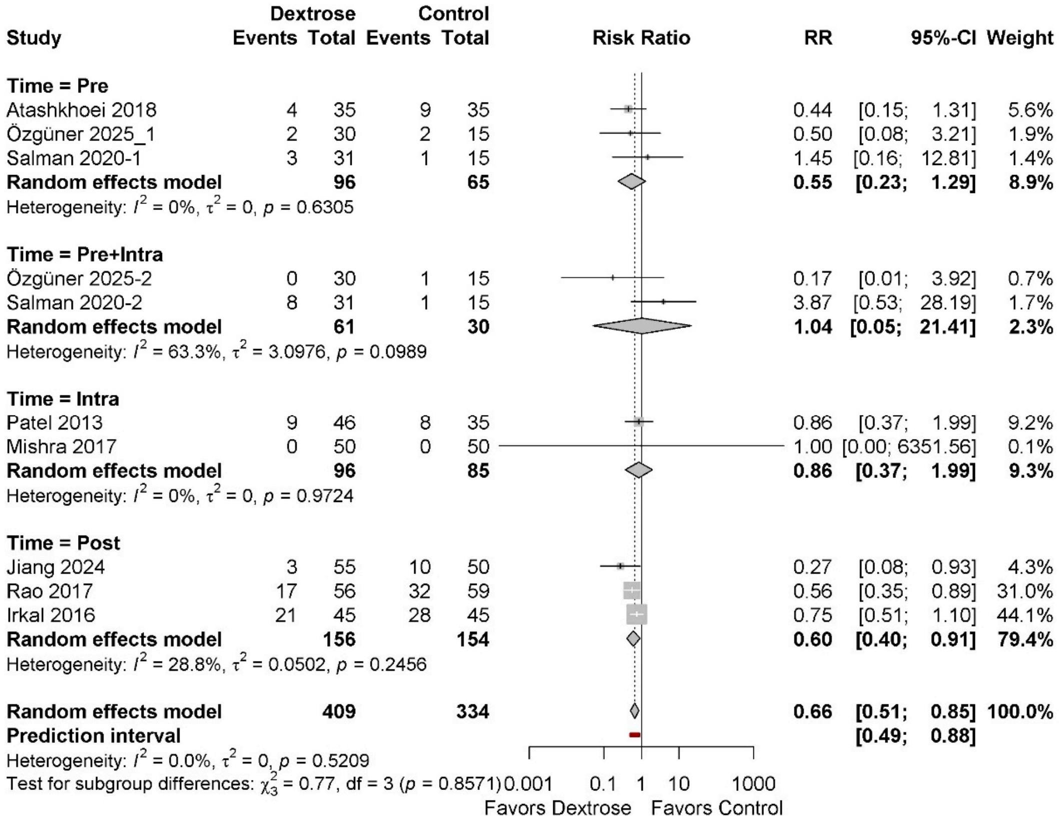

Supplementary Figure S16. Sensitivity analysis removing each study at a time for the incidence of early postoperative nausea and vomiting (PONV). Each study is represented by a filled square proportional to its sample size, with horizontal lines indicating the 95% confidence interval (CI) of the mean difference. The black diamond denotes the overall pooled effect estimate with its 95% CI.

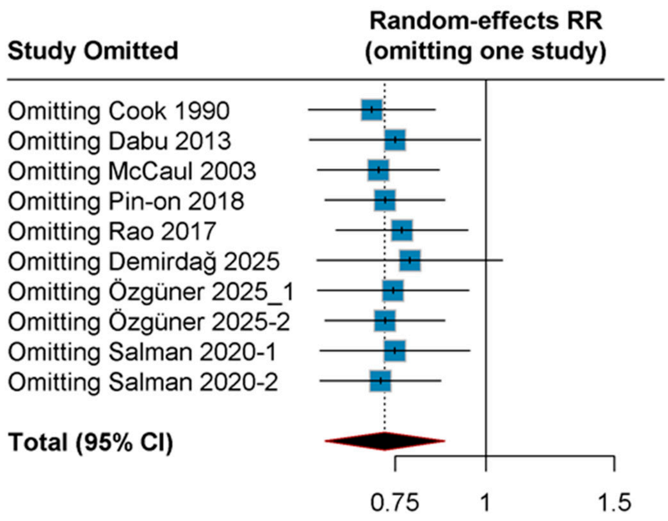

Supplementary Figure S17. Trial sequential analysis (TSA) plot for the incidence of early postoperative nausea and vomiting (PONV). The uppermost and lowermost continuous red lines represent the trial sequential monitoring boundaries for benefit and harm, respectively. The horizontal red lines indicate the conventional boundaries for statistical significance. The cumulative Z-curve (blue solid line) crosses the trial sequential monitoring boundary, indicating that the evidence for the antiemetic effect of dextrose is conclusive**.** The vertical red line on the far right indicates the required information size (RIS) of 1933 patients, and the number 743 on the x-axis represents the total number of patients currently included in the analysis.

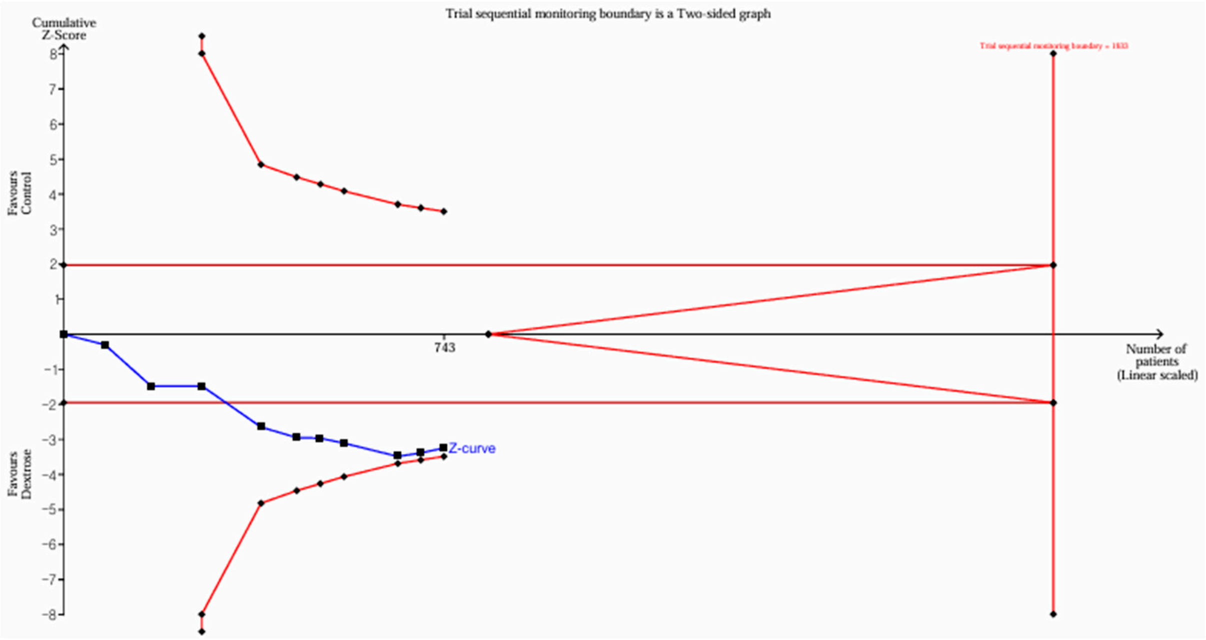

Supplementary Figure S18. Forest plot of the incidence of late postoperative nausea and vomiting (PONV) stratified by the timing of dextrose administration. Each study is represented by a filled square proportional to its weight, with horizontal lines indicating the 95% confidence interval (CI). The black diamonds represent the subgroup-specific pooled risk ratios (RRs), and the open diamond at the bottom indicates the overall pooled RR for all included studies. The red horizontal bar represents the prediction interval. Vertical dashed lines represent the null effect (RR = 1). Heterogeneity was assessed using the I^2 statistic.

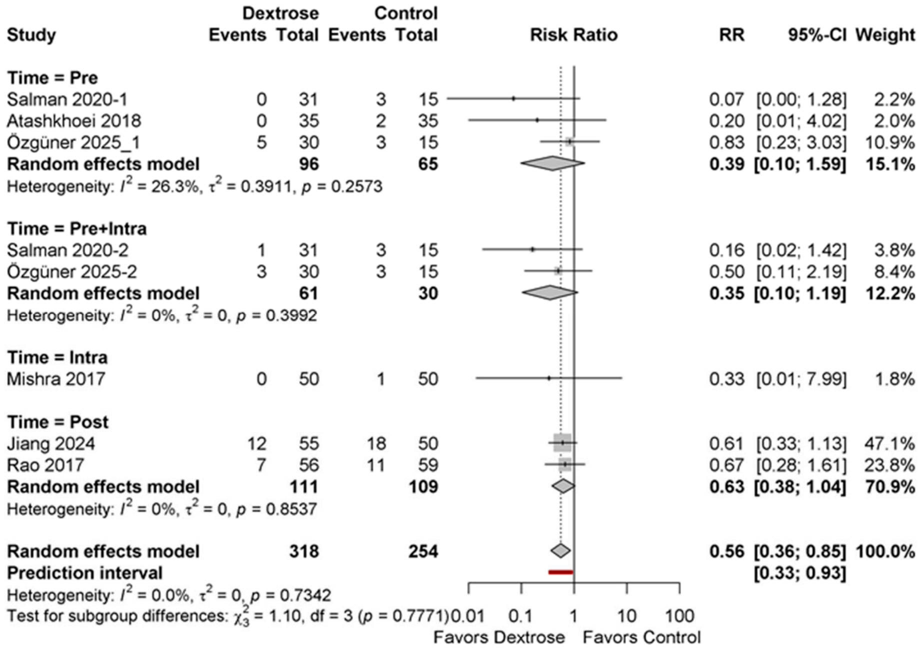

Supplementary Figure S19. Sensitivity analysis removing each study at a time for the incidence of late postoperative nausea and vomiting (PONV). Each study is represented by a filled square proportional to its sample size, with horizontal lines indicating the 95% confidence interval (CI) of the mean difference. The black diamond denotes the overall pooled effect estimate with its 95% CI.

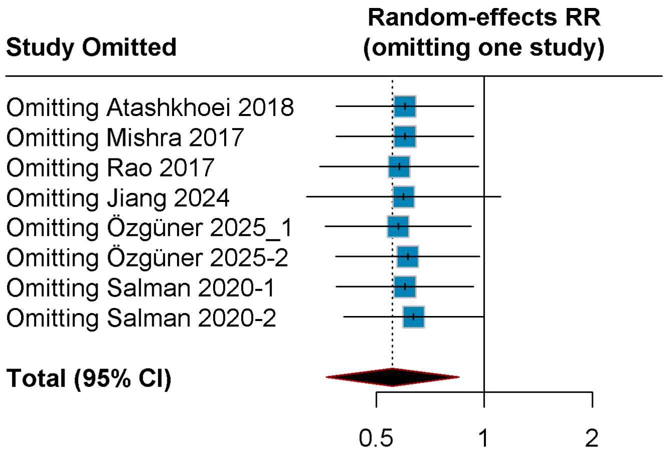

Supplementary Figure S20. Trial sequential analysis (TSA) plot for the incidence of late postoperative nausea and vomiting (PONV). The uppermost and lowermost continuous red lines represent the trial sequential monitoring boundaries for benefit and harm, respectively. The horizontal red lines indicate the conventional boundaries for statistical significance. The cumulative Z-curve (blue solid line) crosses the trial sequential monitoring boundary, indicating that the evidence for the antiemetic effect of dextrose is conclusive. The vertical red line on the far right indicates the required information size (RIS) of 3448 patients, and the number 572 on the x-axis represents the total number of patients currently included in the analysis.

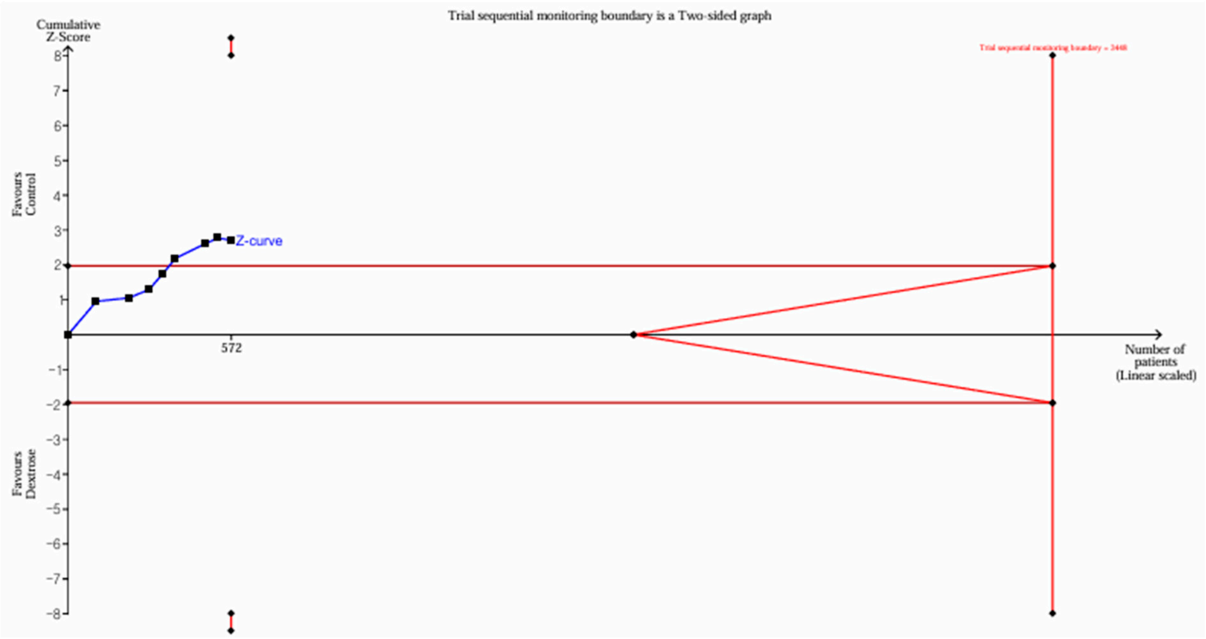

Supplementary Figure S21. Sensitivity analysis removing each study at a time for the incidence of use of rescue anti-emetics. Each study is represented by a filled square proportional to its sample size, with horizontal lines indicating the 95% confidence interval (CI) of the mean difference. The black diamond denotes the overall pooled effect estimate with its 95% CI.

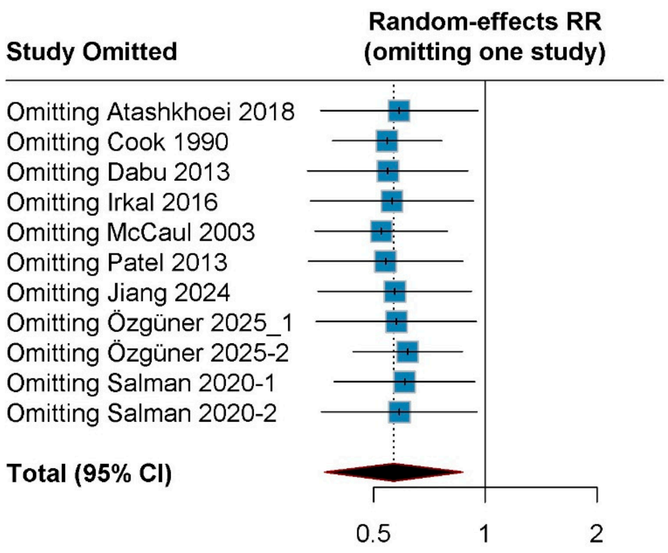

Supplementary Figure S22. Sensitivity analysis comparing frequentist and Bayesian publication bias adjustment methods for the pooled risk ratio of overall postoperative nausea (PON).

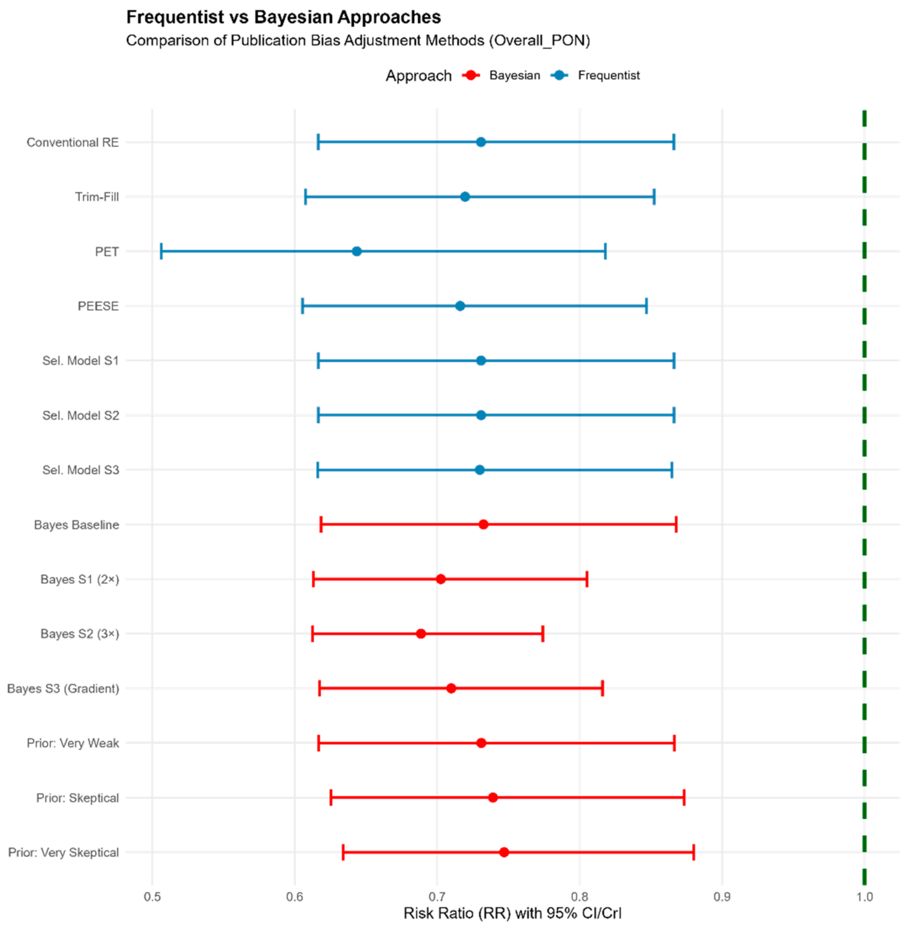

Supplementary Figure S23. Sensitivity analysis comparing frequentist and Bayesian publication bias adjustment methods for the pooled risk ratio of overall postoperative nausea and vomiting (PONV).

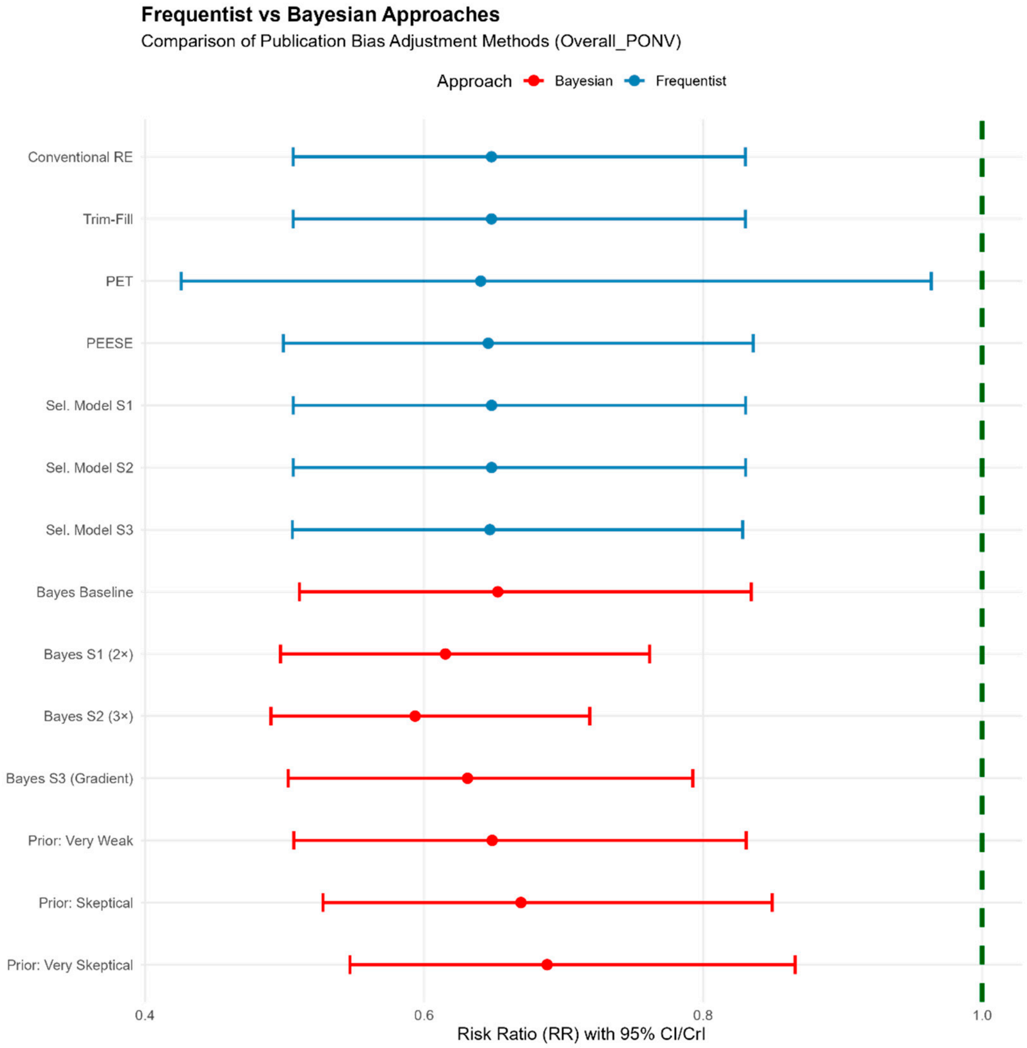

Supplementary Figure S24. Sensitivity analysis comparing frequentist and Bayesian publication bias adjustment methods for the pooled risk ratio of overall postoperative vomiting (POV).

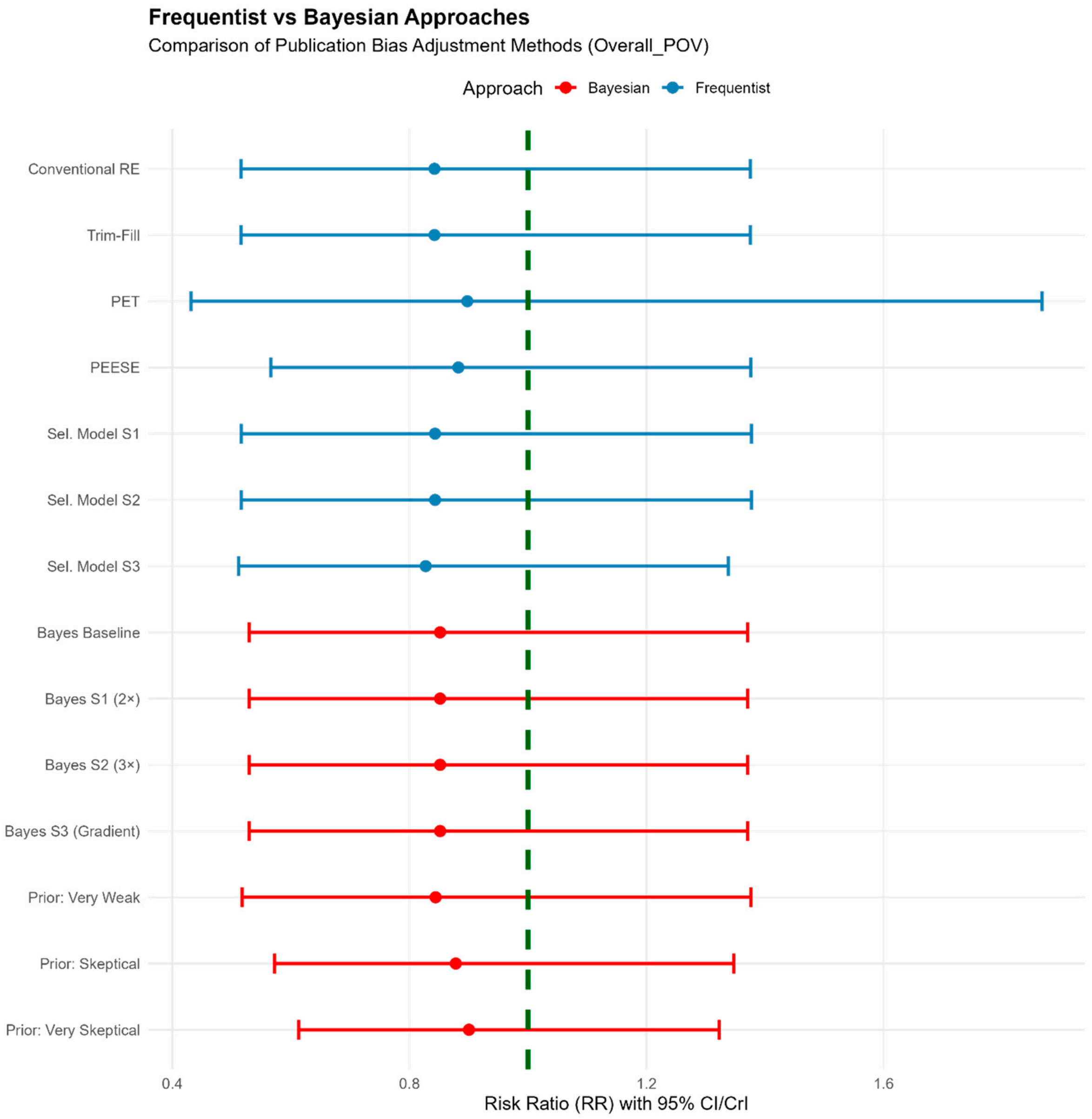

Supplementary Figure S25. Sensitivity analysis comparing frequentist and Bayesian publication bias adjustment methods for the pooled risk ratio of early postoperative nausea (PON).

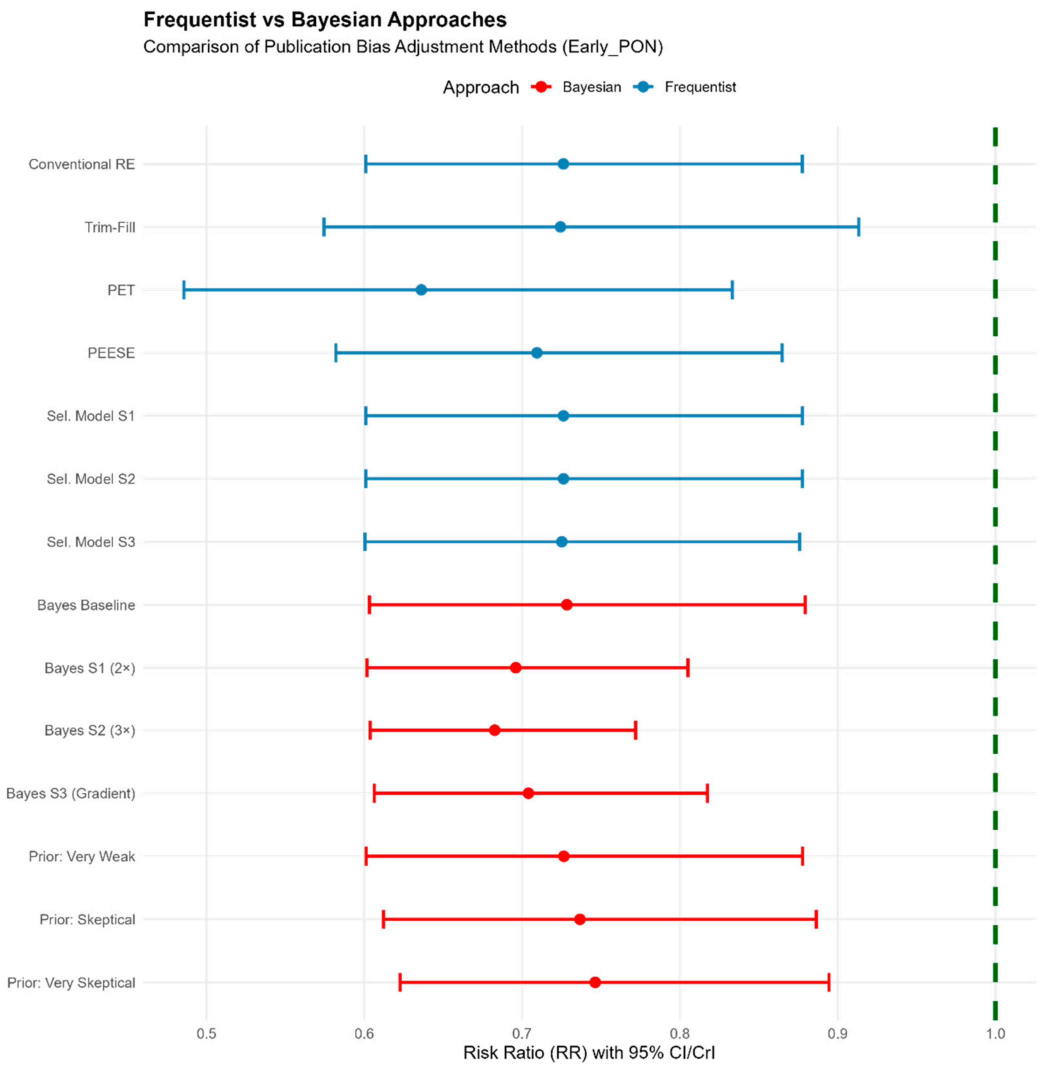

Supplementary Figure S26. Sensitivity analysis comparing frequentist and Bayesian publication bias adjustment methods for the pooled risk ratio of early postoperative nausea and vomiting (PONV).

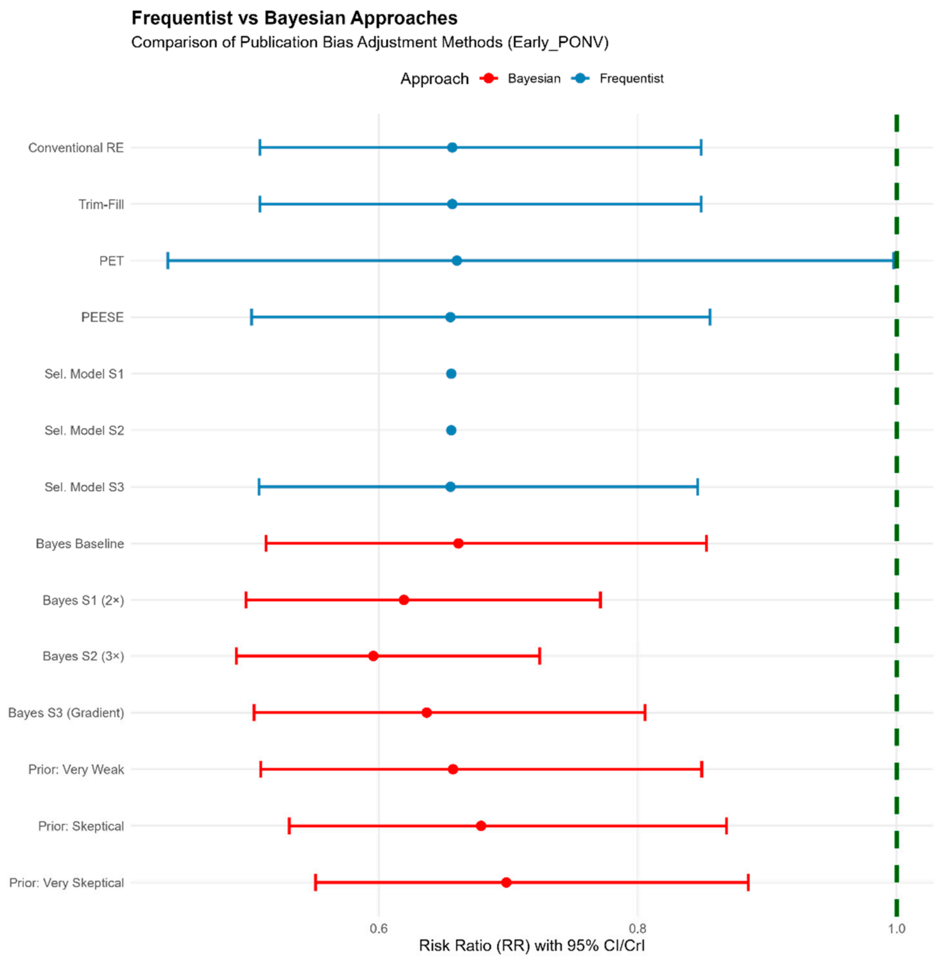

Supplement: Supplementary file 1 [file medicina-62-01718-s001.zip › medicina-4442420-supplementary.pdf]
